# MiR-423-5p is a metabolic and growth tuner in hepatocellular carcinoma via MALAT-1 and mitochondrial interaction

**DOI:** 10.1186/s13046-025-03524-2

**Published:** 2025-09-30

**Authors:** Marco Bocchetti, Alessia Maria Cossu, Manuela Porru, Maria Grazia Ferraro, Carlo Irace, Rossella Tufano, Giovanni Vitale, Gabriella Misso, Nicola Amodio, Marianna Scrima, Ines Simeone, Michele Ceccarelli, Ugo Chianese, Lucia Altucci, Vincenzo Desiderio, Tarik Regad, Michele Caraglia, Silvia Zappavigna

**Affiliations:** 1 Molecular Oncology and Precision Medicine Laboratory, Biogem IRGS, Contrada Camporeale, Ariano Irpino, 83031 AV Italy; 2 Department of Life Sciences, Health and Health Professions, Link Campus University, Via del Casale di San Pio V 44, Rome, 00165 RM Italy; 3 Department of Precision Medicine, University of Campania “Luigi Vanvitelli”, Via De Crecchio, Naples, 80131 NA Italy; 4 John van Geest Cancer Research Centre, Nottingham Trent University, Clifton Lane, Nottingham, NG121AS Clifton United Kingdom; 5 Translational Oncology Research Unit, IRCCS - Regina Elena National Cancer Institute, Via Elio Chianesi 53, Rome, 00144 RM Italy; 6 Department of Molecular Medicine and Medical Biotechnology, University of Naples “Federico II”, Via Pansini, Naples, 80131 NA Italy; 7 Department of Pharmacy, University of Naples “Federico II”, Via Domenico Montesano, Naples, 80131 NA Italy; 8 Laboratory of Computational Biology, Biogem IRGS, Contrada Camporeale, Ariano Irpino, 83031 AV Italy; 9 Department of Electrical Engineering and Information Technologies, University of Naples “Federico II”, Naples, NA Italy; 10 Laboratory of Geriatric and Oncologic Neuroendocrinology Research, IRCCS, Istituto Auxologico Italiano, Milan, 20122 MI Italy; 11 Department of Medical Biotechnology and Translational Medicine, University of Milan, Milan, 20122 MI Italy; 12 Department of Experimental and Clinical Medicine, Magna Graecia University of Catanzaro, Viale Europa, Catanzaro, 88100 CZ Italy; 13 Humanitas Research Hospital, Via Manzoni, Rozzano, 20089 MI Italy; 14 Department of Public Health Sciences, Miller School of Medicine, University of Miami, Miami, Florida USA; 15 Department of Experimental Medicine, University of Campania “Luigi Vanvitelli”, Via De Crecchio, Naples, 80131 NA Italy

**Keywords:** MicroRNA, HCC, Mitochondria, Metabolism, MiR-423-5p, MALAT-1

## Abstract

**Background:**

MicroRNAs (miRNAs) and long non-coding RNAs (lncRNAs) are key regulators of gene expression and play a crucial role in cancer progression. Recent studies have highlighted miR-423-5p as a potential modulator in hepatocellular carcinoma (HCC), especially in patients responding to sorafenib treatment. A functional interaction with the oncogenic lncRNA MALAT-1 has been hypothesized, suggesting a regulatory mechanism that may influence tumor aggressiveness.

**Methods:**

To investigate this interaction, we analyzed in silico patient datasets to correlate miR-423-5p and MALAT-1 expression with overall survival (OS) and disease free survival (DFS). Stable overexpression of miR-423-5p and MALAT-1 was achieved in HCC cell lines (HepG2, Hep3B, and SNU387) using a lentiviral transduction system. Functional assays were performed to assess proliferation, migration, invasion, and clonogenic potential. The interaction between miR-423-5p and MALAT-1 was confirmed by RNA immunoprecipitation (RIP), followed by transcriptomic analysis using next-generation sequencing (NGS). Mitochondrial activity was evaluated using the Seahorse Mito Stress Test to measure oxygen consumption rate (OCR) and ATP production. In vivo experiments in orthotopic mouse models were performed to assess tumor growth.

**Results:**

Patient data analysis revealed that high miR-423-5p expression correlated with a less aggressive tumor phenotype and improved survival, while MALAT-1 was associated with poorer prognosis. In vitro, miR-423-5p overexpression reduced MALAT-1 levels and significantly impaired proliferation, migration, and invasion. NGS analysis identified transcriptomic changes linked to tumor progression and metabolic shift. The Seahorse Mito Stress Test demonstrated decreased cellular respiration and ATP production upon miR-423-5p overexpression. In vivo, both tumors derived from miR-423-5p-overexpressing cells and MALAT-1 downregulation by ASO GapmeR evidenced a significantly reduced growth compared to controls.

**Conclusion:**

These findings suggest, for the first time, that miR-423-5p acts as a tumor suppressor affecting mitochondrial metabolism through MALAT-1 downregulation in HCC. This regulatory axis represents a potential therapeutic target for precision medicine approaches in liver cancer.

**Supplementary Information:**

The online version contains supplementary material available at 10.1186/s13046-025-03524-2.

## Background

Liver cancer, and especially hepatocellular carcinoma (HCC), is still among the most widespread and aggressive types of cancer across the world [1]. It often has a poor prognosis due to its late detection and the few treatment options available. Many patients also stop responding to commonly used drugs like sorafenib and lenvatinib, making treatment even more difficult [2, 3]. On this basis, there is an urgent need to find new molecular targets and biomarkers that can improve therapies and help predict which treatments might be effective [4, 5]. The disease complexity and the inter-patient variation add another layer of difficulty, stressing the importance of new, creative research that uncovers how liver cancer progresses on a molecular level [6]. In recent years, scientists worldwide have shown how small RNA molecules like microRNAs (miRNAs) and long non-coding RNAs (lncRNAs) are crucial in cancer development. These molecules play a role in many aspects of tumors, such as how they grow, spread, manage energy, and develop resistance to drugs [7–10]. One specific miRNA, called miR-423-5p, has caught researchers’ attention because it seems to suppress tumors in several cancers. Higher levels of miR-423-5p have been linked to tumor growth inhibition and to reduced invasiveness of ovarian, colon, and breast cancers. This points to its role in promoting cell death and stopping metastasis [11, 12]. Moreover, our previous work showed that miR-423-5p could control prostate cancer growth and invasiveness by interacting with a known cancer-related non-coding RNA, MALAT-1, which altered cell metabolism and progression [13]. MALAT-1, short for Metastasis-associated lung adenocarcinoma transcript 1, is well-known for fueling cancer. It is often found at high levels in many solid tumors and is linked to poor outcomes, helping cancer cells to grow, spread, and resist to treatment [14, 15]. The latter effects are induced by the modulation of important internal cell signaling pathways related to metabolism and metastasis. This happens mainly through its ability to bind with specific proteins and miRNAs [16, 17]. Recent research also shows that MALAT-1 plays a role in changing how cancer cells use energy, improving mitochondrial function, and helping cells to survive upon stress and chemotherapy [18, 19]. Even though there is growing evidence of their importance, the specific connection between miR-423-5p and MALAT-1 in liver cancer has not been deeply studied yet. Interestingly, some early findings suggest this interaction could be of pivotal importance, both biologically and clinically. For example, our previous studies showed that liver cancer patients who responded to sorafenib had higher levels of circulating miR-423-5p, which was associated with the autophagy cell death pathways in vitro [20]. These results support the idea that the miR-423-5p and MALAT-1 relationship could play a big role in how liver cancer cells manage energy and respond to treatments. Given this background, our current study takes a close look at how miR-423-5p and MALAT-1 work together in liver cancer, focusing specifically on how they influence tumor functionality and mitochondrial metabolism. We have used a combination of approaches, ranging from computer-based data analysis and stable cell-line engineering to full transcriptome analysis, mitochondrial activity tests, and animal models of liver cancer. Our study aims to clarify the regulatory axis between miR-423-5p and MALAT-1 and to determine whether miR-423-5p exerts tumor-suppressive effects in HCC by influencing mitochondrial metabolism. Validation of the anti-tumor effectiveness of both miR-423-5p replacement and MALAT-1 downregulation through LNA Antisense GapmeR delivery was assessed in an in vivo model of HCC. The understanding of these interactions could provide new perspectives for targeted therapeutic approaches in liver cancer [21].

## Results

### miR-423-5p and MALAT-1 in HCC patients

**Fig. 1:**
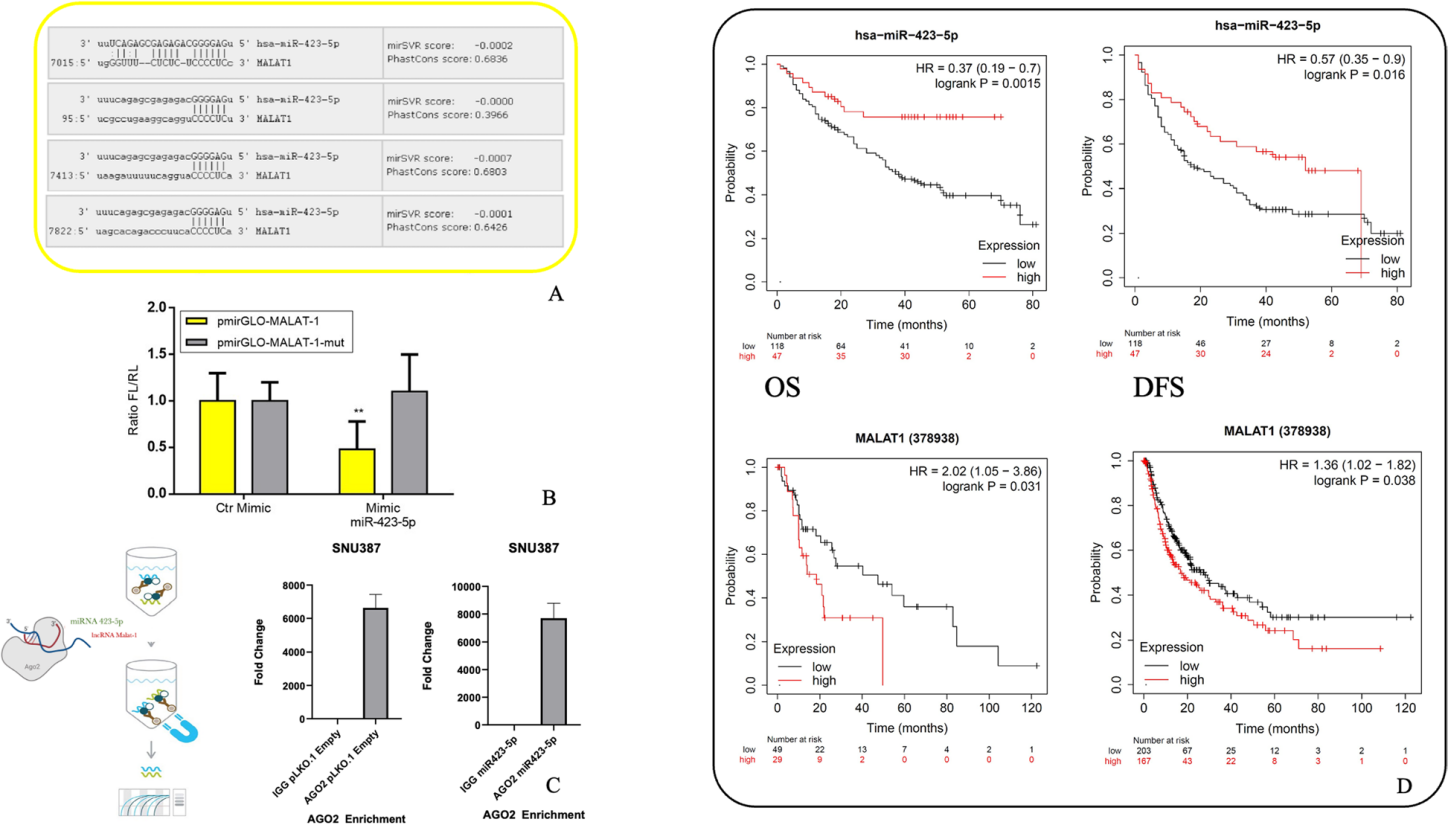
miR-423-5p targets MALAT-1 through direct interaction and correlates with survival outcomes in HCC patients. **A** In silico prediction of miR-423-5p binding sites on the MALAT-1 transcript using TargetScan. Two conserved regions (binding sites A and B), located at positions 94 and 7413 respectively, were identified based on PhastCons scores <0.3. **B** Dual-luciferase reporter assay showing that co-transfection of miR-423-5p mimic with the wild-type MALAT-1 3’ UTR construct in HepG2 cells significantly reduces luciferase activity compared to control mimic, while no effect is observed with a mutated reporter construct. **C** RNA immunoprecipitation (RIP) assay using anti-AGO2 antibody reveals the presence of both miR-423-5p and MALAT-1 in the AGO2 complex, confirming physical interaction. FOS mRNA was used as a positive control; IgG antibody served as a negative control. **D** Kaplan-Meier survival curves for overall survival (OS) and disease-free survival (DFS) in hepatocellular carcinoma patients. * $p < 0.05$, ** *p*-value $< 0.01$, *** *p*-value $< 0.001$, **** *p*-value $< 0.0001$

One of lncRNAs mode of action is the regulation of miRNAs expression that, in turn, can modulate lncRNAs expression through their degradation [22]. We have previously reported that miR-423-5p induces anti-metastatic effects in prostate cancer cells by downregulation of the lncRNA MALAT-1 physically interacting with the latter in vitro [13]. On these bases, here we used TargetScan software in order to predict in silico the binding sites of miR-423-5p and MALAT-1. We identified four distinct binding sites between the ribonucleotide sequence of lncRNA MALAT-1 and hsa-miR-423-5p. We then narrowed our selection to binding sites with a PhastCons score exceeding 0.3 (the default value), leading us to identify the binding sites A and B as the most relevant (Fig. 1A). These two distinct regions within MALAT-1 were found to be placed at positions 94 and 7413, respectively. Interestingly, binding site B, located in the Terminal domain of MALAT-1, plays a crucial role in the stability and half-life of the lncRNA, as suggested by previous studies [23]. To investigate the direct binding of miR-423-5p to MALAT-1, we performed dual-luciferase assays. We employed pmirGLO-MALAT-1 (wild type) and pmirGLO- MALAT-1-Mut luciferase reporter vectors, each containing the targeted sequences, along with either miR-423-5p Mimic or a Control (Ctr). These constructs were separately transfected into HepG2 cells. When the wild-type reporter vector for MALAT-1 was co-transfected with miR-423-5p Mimic, there was a significant reduction in relative luciferase activity compared to the wild-type vector co-transfected with the Control Mimic (Fig. 1B). In contrast, no reduction was observed when the mutated vector was used in combination with miR-423-5p Mimic. These findings strongly suggested that miR-423-5p likely exerts its influence on MALAT-1 mRNA expression at the post-transcriptional level (Fig. 1B). Additionally, we performed RNA Immunoprecipitation with the use of the AGO2 RNA-Binding antibody for enrichment. As shown in Fig. 1D, both miR-423-5p and MALAT-1 were identified in the AGO2 immunoprecipitate, and this confirmed their physical interaction at the AGO2 level. To validate the AGO2 enrichment, we employed FOS primers. We compared these results to a control experiment that used a negative control IgG antibody (Fig. 1C). To establish a robust clinical context for these non-coding RNAs, we investigated patient clinical data, including survival outcomes. Survival analysis and Kaplan-Meier curves for Overall Survival (OS) and Disease-Free Survival (DFS) were generated using the Kaplan-Meier Plotter online tool (KM Plotter, http://kmplot.com/analysis), which integrates publicly available transcriptomic datasets from liver cancer patients. Therefore, we generated survival curves based on the expression of miR-423-5p and MALAT-1 and examined OS and DFS. The results (Fig. 1D), demonstrated that increased miR-423-5p expression correlates with a more favorable prognosis, as indicated by a Hazard Ratio (HR) coefficient of less than 0.6 and significant *p*-values. These findings were consistent across a dataset of over 100 patients for both OS and DFS. Conversely, the increased expression of MALAT-1 was associated with a significant decrease in survival rates, characterized by an HR exceeding 1.36 and significant *p*-values. This suggested that miR-423-5p was indicative of a milder cancer phenotype and appeared to confer a protective effect in HCC patients. In contrast, the lncRNA MALAT-1 caused an opposing influence, exacerbating cancer conditions and contributing to poorer outcomes.

### Transient miR-423-5p overexpression on HCC cell lines

**Fig. 2:**
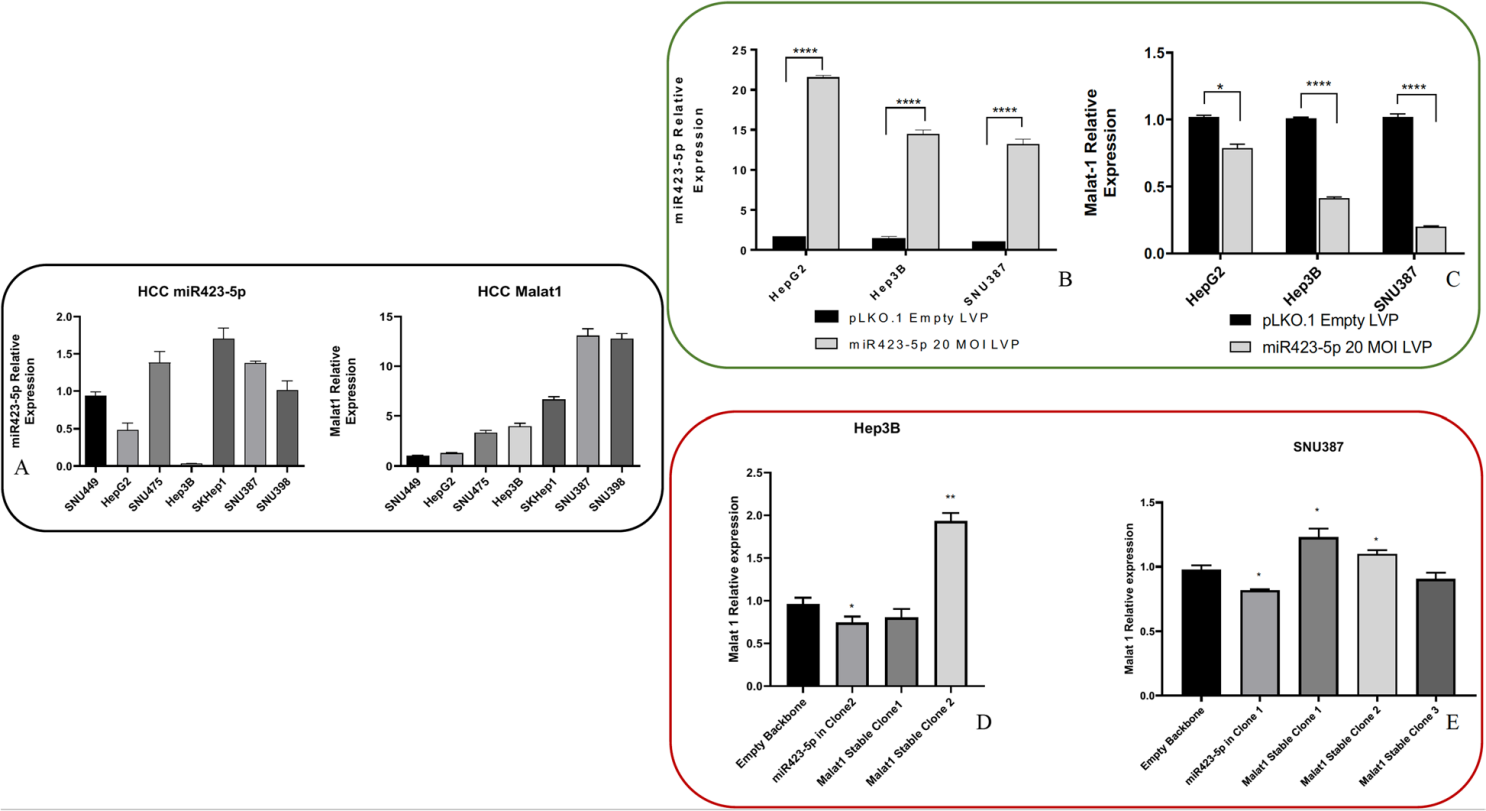
**A** Basal expression levels of miR-423-5p and MALAT-1 across seven HCC cell lines (SNU449, HepG2, SNU475, Hep3B, SKHep1, SNU387, and SNU389) assessed by qRT-PCR. Expression values were normalized to endogenous controls and used to guide selection of models for functional assays. **B** Generation of stable miR-423-5p-overexpressing clones in HepG2, Hep3B, and SNU387 cells using lentiviral vectors expressing GFP. Puromycin selection was applied to enrich successfully transduced cells. qRT-PCR confirmed persistent miR-423-5p upregulation. **C** Stable overexpression of miR-423-5p led to a consistent downregulation of MALAT-1 levels compared to vector-only controls in all three cell models. **D**–**E** Establishment of stable MALAT-1-overexpressing clones in SNU387 and Hep3B cells via lentiviral transduction. Overexpression was confirmed by qRT-PCR. In the selected clones (SNU387 clone 1 and Hep3B clone 2), MALAT-1 upregulation was associated with reduced miR-423-5p levels, confirming a reciprocal regulatory relationship. * $p < 0.05$, ** *p*-value $< 0.01$, *** *p*-value $< 0.001$, **** *p*-value $< 0.0001$

Based on the previous results, we have investigated upon the reciprocal regulation of the expression of miR-423-5p and MALAT-1 in HCC cell lines. Firstly, we have evaluated miR-423-5p and MALAT-1 expression levels in 7 different HCC cell lines: SNU449, HepG2, SNU475, Hep3B, SKHep1, SNU387, and SNU389, using real-time PCR analysis (Fig. 2A). This initial investigation was crucial for selecting appropriate in vitro models for our subsequent studies. Specifically, we selected SNU387, characterized by notably high MALAT-1 expression, Hep3B, which exhibited the lowest miR-423-5p expression, and HepG2, a well-established in vitro model for HCC. To explore the functional role of miR-423-5p in regulating HCC cell behavior, we performed transient transfections on HepG2, Hep3B, and SNU387 cells using Mimic miR-423-5p or miR-423-5p Inhibitor. We assessed the impact on miRNA levels using RT-qPCR. Our results revealed a substantial increase in miR-423-5p expression 72 hours after transfection with Mimic, whereas Inhibitor effectively reduced miR-423-5p levels (Supplementary Fig. 1). Subsequently, we examined the expression of MALAT-1 through qRT-PCR to investigate its potential interaction with miR-423-5p. Encouragingly, our findings indicated that transient overexpression of miR-423-5p led to the downregulation of MALAT-1, while its downregulation resulted in MALAT-1 expression levels comparable to or higher than controls in all our cellular models, validating our hypothesis (Supplementary Fig. 1). Notably, the miR-423-5p inhibitor was also capable of reverting the observed phenotype, as a proof of concept (Supplementary Fig. 1).

### Stable miR-423-5p or MALAT-1 transfection induce reciprocal expression regulation

To explore the prolonged impact of miR-423-5p overexpression in HCC cell lines, we generated stable overexpressing clones along with matched controls using a lentiviral transduction system (Fig. 2B). To ensure the survival of only the transduced cells, we subjected them to puromycin selection. These cells were regularly monitored for the presence of the GFP reporter gene, which served as a marker indicating miR-423-5p expression (Supplementary Fig. 2). The upregulation of miR-423-5p expression was confirmed via qRT-PCR analysis, demonstrating a significant increase in the stably transfected cells compared to the control group. We used lentiviruses with various MOIs (5, 10, 15, 20) for the transduction of HCC cell lines. The efficiency of transduction was assessed by observing GFP fluorescence under a microscope, beginning 24 hours after infection (Supplementary Fig. 2). To verify the overexpression of miR-423-5p and its impact on the target lncRNA MALAT-1, we evaluated both expression levels in overexpressing clones and compared them with respective controls. Our findings indicated that the expression of MALAT-1 decreased in response to miR-423-5p overexpression in all the three cell models when compared to the matching empty vector models (Fig. 2C). Subsequently, we selected and amplified the most promising clones transduced with a MOI of 20 for additional in vitro experiments. In a complementary experiment, we transiently transfected HepG2 cells, which stably overexpressed miR-423-5p, with a synthetic oligonucleotide designed to inhibit miR-423-5p (Inhibitor). The results of this proof of concept experiment additionally supported our hypothesis: in our miR-423-5p overexpressing cell line, the subsequent inhibition of miRNA led to a significant increase in MALAT-1 levels (Supplementary Fig. 3). To additionally confirm MALAT-1 effects on cancer cell lines the two more promising in vitro models, Hep3B and SNU387, were selected to establish MALAT-1 overexpressing clones and relative controls using the same lentiviral transduction approach. Three separate infection protocols were used to obtain SNU387 clones and two to obtain Hep3B clones. The overexpression was then confirmed by qRT-PCR analysis, and the best results were obtained in SNU387 clone 1 and Hep3B clone 2 compared to the controls (Fig. 2D, E) and the latter were selected for additional experiments. Interestingly, the MALAT-1 overexpressing models showed decreased miR-423-5p levels, confirming the inverse correlation between the two different non-coding RNAs.

### miR-423-5p overexpression inhibited HCC cancer cell growth, while MALAT-1 overexpression had no effect

**Fig. 3:**
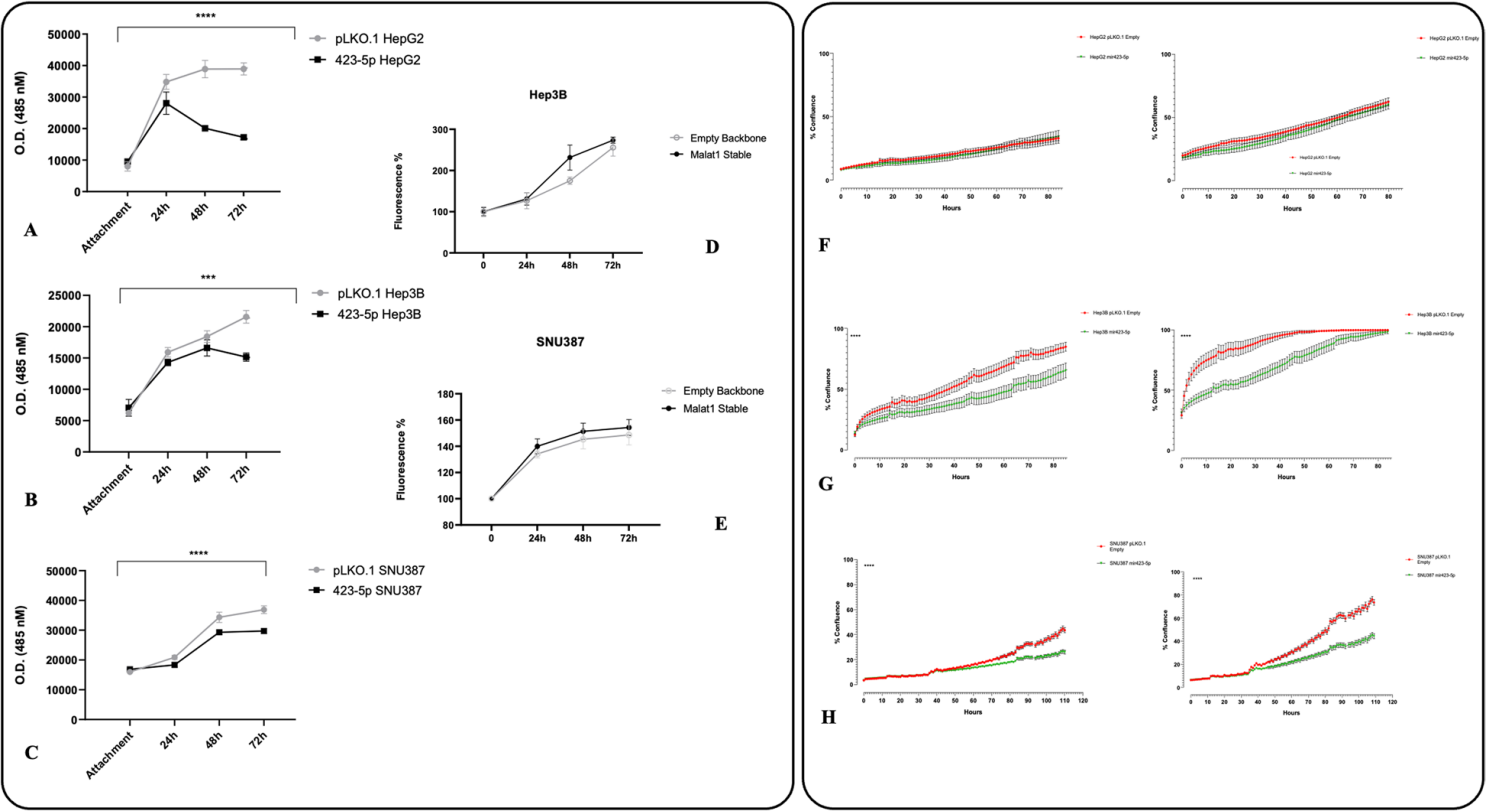
miR-423-5p overexpression reduces proliferation in HCC cell lines (**A**-**C**), while MALAT-1 overexpression alone does not affect growth (**D**-**E**). Live-cell monitoring of cell confluence using the IncuCyte system in Hep3B and SNU387 models seeded at two different densities (**F**–**H**). miR-423-5p-overexpressing cells exhibited markedly reduced confluence compared to control cells over time. * $p < 0.05$, ** *p*-value $< 0.01$, *** *p*-value $< 0.001$, **** *p*-value $< 0.0001$

We and others have suggested that miR-423-5p acts as a tumor suppressor in different types of cancers [13, 24, 25]. Consequently, we investigated how this microRNA was involved in the regulation of growth and proliferation of our HCC miR-423-5p transduced stable models. To do so, we employed the CyQUANT DNA assay in HepG2, Hep3B, and SNU387 cells at different time points. Over time, we observed a reduction in cell proliferation when miR-423-5p was overexpressed, in contrast to the control groups (Fig. 3A, B, C). In addition to the CyQUANT assay, we used the IncuCyte live-cell analysis system to further study the proliferation of these cell lines. Inside the incubator, plates containing miR-423-5p overexpressing cells were scanned and photographed to assess the percentage of confluence and the subsequent increase in proliferation over time, comparing it to the matching empty backbone transduced cells. We assessed two different cell densities for each cell line, and the results are represented in the graphs in Fig. 3F, G and H. Remarkably, we observed a substantially lower percentage of confluence and proliferation in miR-423-5p overexpressing Hep3B and SNU387 cells. On the other hand, we were not able to evaluate proliferation of HepG2 cells as this cell line grows in clusters. Nevertheless, we did observe that these clusters were significantly denser in the control cell line compared to the miR-423-5p overexpressing cell lines, as supported by live-time video footage (not shown) and the CyQuant Assay data mentioned earlier. Interestingly, while looking at the live-time videos, we noticed important differences in shape, growing pattern and intracellular organelles compartmentalization, stimulating metabolism involvement questions. As you can see, MALAT-1 overexpression alone was not able to act specifically on the HCC cell lines proliferation (Fig. 3D, E).

### miR-423-5p mediated MALAT-1 downregulation inhibited HCC cells migration, invasion and clonogenicity potential

**Fig. 4:**
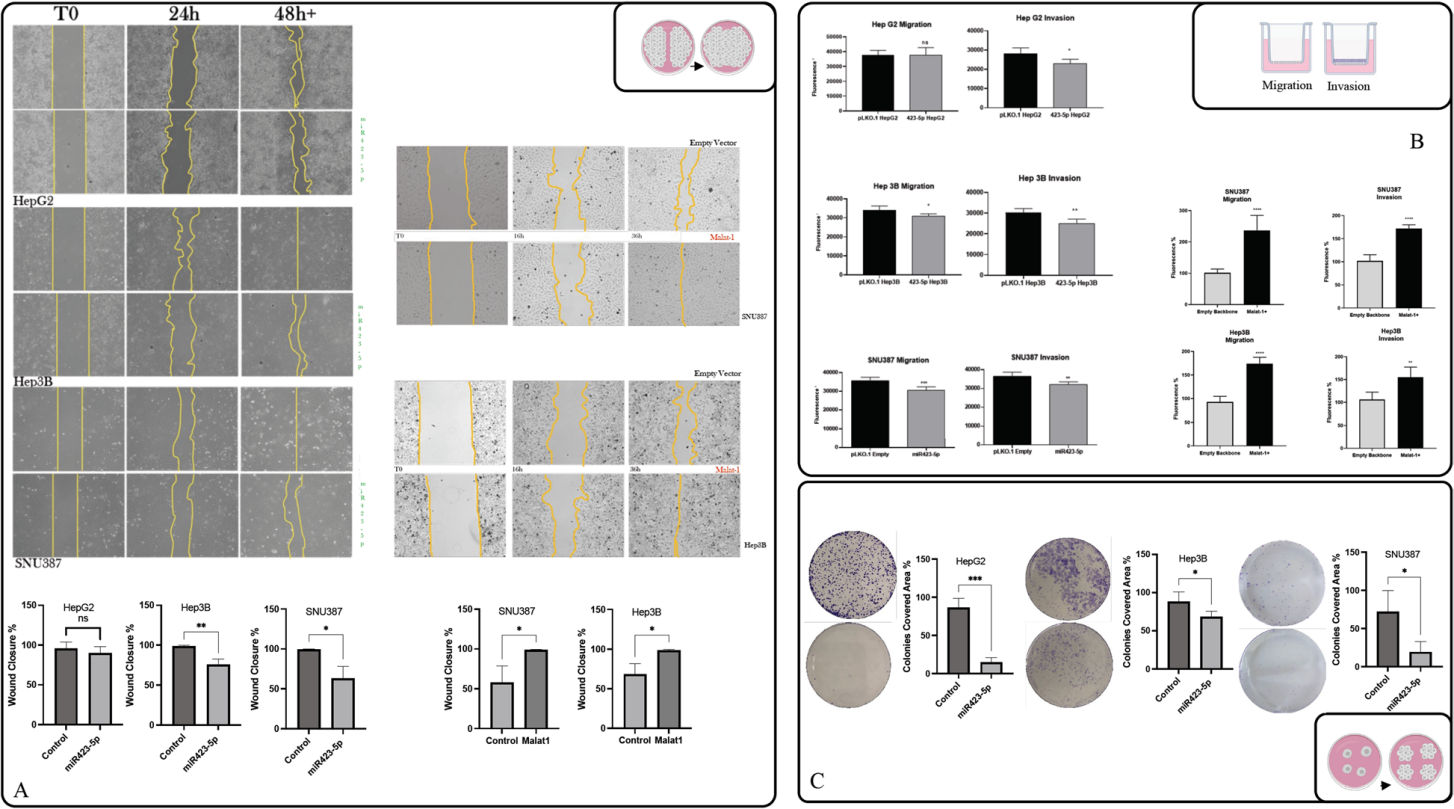
**A** Wound healing assay performed in HepG2, Hep3B, and SNU387 cell lines stably overexpressing miR-423-5p vs empty vector (Left side). Wound healing assay performed in Hep3B, and SNU387 cell lines stably overexpressing MALAT-1 vs empty vector (Right side). **B** Transwell migration and invasion assay using Cultrex BME-coated inserts. miR-423-5p overexpression significantly decreased the number of migrating and invading cells after 48 hours compared to control cells (Left Side). MALAT-1 overexpression is remarkably boosting migrating and invasive capability of HEP3B and SNU387 (Right side). **C** Colony formation assay under low-density and semi-starved conditions for 14 days revealed impaired clonogenicity in all miR-423-5p-overexpressing HCC cell models compared to respective controls. * $p < 0.05$, ** *p*-value $< 0.01$, *** *p*-value $< 0.001$, **** *p*-value $< 0.0001$

Given the established link between elevated MALAT-1 expression and metastasis, we investigated the potential impact of miR-423-5p-induced MALAT-1 down-regulation on the migratory and invasive properties of HepG2, Hep3B, and SNU387 HCC cell lines. We performed migration assessment experiments using uncoated Cultrex BME plates and wound healing assays. The results, as shown in Fig. 4, clearly indicated that the migration ability of the HCC cell lines was significantly reduced in cells stably overexpressing miR-423-5p, as compared to those transfected with the empty vector. This reduction is distinctly visible in the images captured during the wound healing assay (Fig. 4A). Moreover, we observed a notable reduction in invasion towards FBS in a Cultrex BME matrix-coated cell invasion assay after 48 hours (Fig. 4B). To assess the self-renewal and clonogenic potential of those models, we employed the Colony Formation Assay, comparing the three overexpressing cell lines to their respective controls. The panel in the Fig. 4C shows the difference in colony formation, revealing impaired colony formation in the miR-423-5p overexpressing cell lines after 14 days of incubation under conditions of low density and semi-starvation.

### MALAT-1 mediated miR-423-5p downregulation increased HCC cells migration, invasion and clonogenicity potential

After the evaluation of miR-423-5p influence on HCC cell lines functional phenotype, we assessed the effects of MALAT-1 overexpression as well, in order to clarify the role of the latter in our specific HCC models. Using similar approaches we studied MALAT-1 upregulation effects on proliferation, migration, invasion and clonogenic potential on Hep3B and SNU387 clone 2 and clone 1, respectively. The DNA dye CyQuant NF fluorescence was measured at different time points (Cell attachment, 24 h, 48 h, 72 h) to measure the DNA amount reflecting the proliferation. MALAT-1 overexpressing clones’ proliferation was compared with the relative empty backbone control and shown in Fig. 3D and E. No significant modifications in cell proliferation were observed after 72 h in this case, even if MALAT-1 seems to slightly boost the proliferation. On the other hand, we obtained remarkable and significant changes on migration and invasion. Migration was assessed by wound healing assay for both cell lines (Fig. 4A). Trevigen Cultrex BME 96 well plate was used for both the invasion and migration assays with a membrane coated with BME collagen-like matrix and with uncoated membrane, respectively (Fig. 4B). In those Hep3B and SNU387 models, migration and invasion were significantly increased after MALAT-1 overexpression.

### Whole transcriptome analysis of MALAT-1 and miR-423-5p transfected HCC cells

**Fig. 5:**
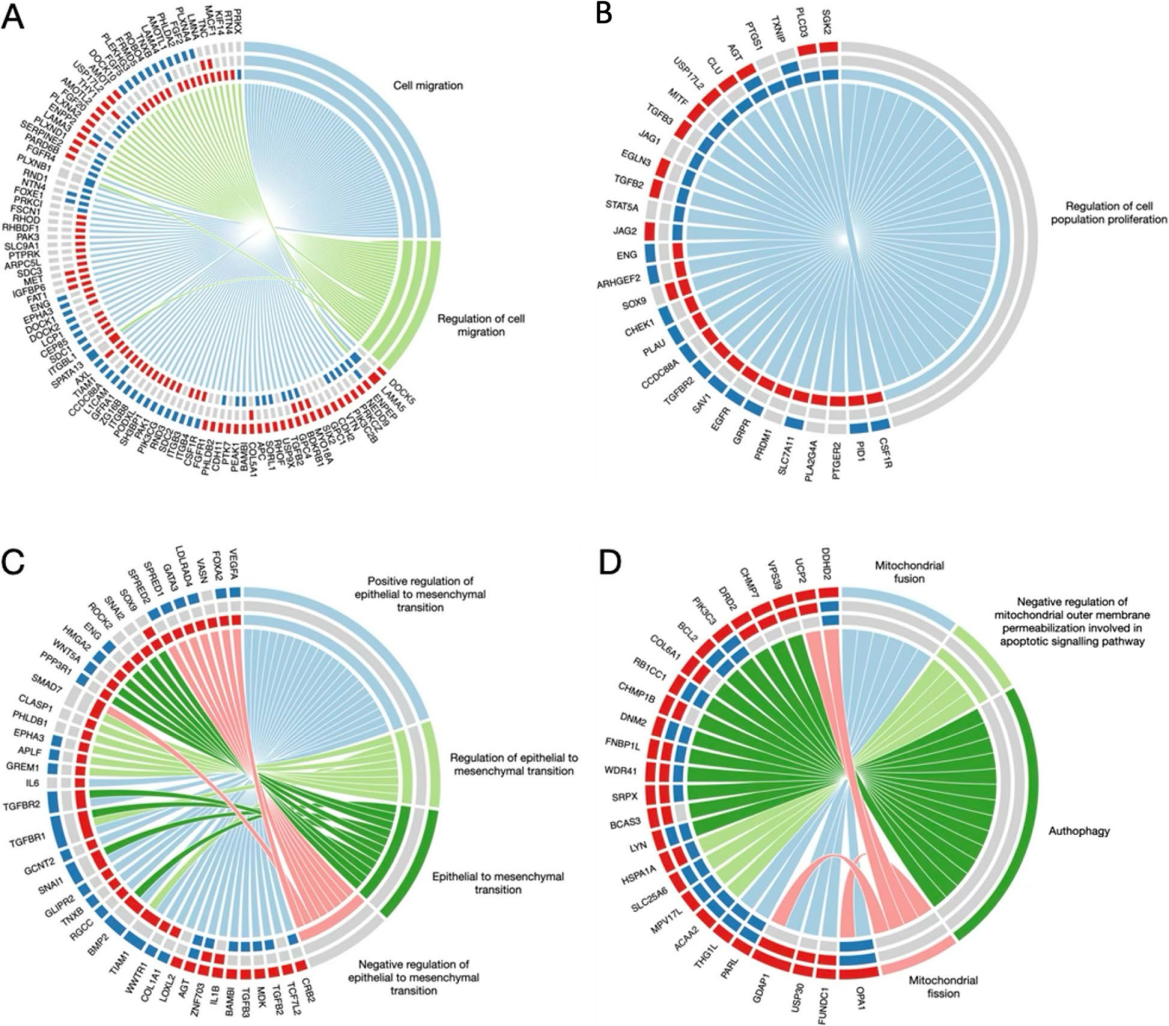
The circos plots show the selected biological processes (BPs) related to **A** cell movement, **B** cell proliferation, **C** epithelial to mesenchymal transition, and **D** mitochondria and autophagy. Each circos plot is composed of three tracks that show the enriched BPs and the related genes identified by comparing MALAT-1 transduced cells vs miR-423-5p transduced cells (external track), MALAT-1 transduced cells vs the cells infected with empty vector (middle track) and miR-423-5p transduced cells vs the cells infected with empty vector (inner track). The rectangles representing the genes are red, blue, or grey to indicate the up-regulated, down-regulated, and not significant genes identified for each comparison. The rectangles representing the BPs are grey for *p*-value $< 0.05$

The expression profiles of SNU-387 cell lines transfected with miR-423-5p ($n = 2$), MALAT-1 ($n = 2$), and empty vector ($n = 2$) were investigated to identify dysregulated genes involved in cell movement, proliferation, and invasion. We first confirmed the established modifications: the cell lines transduced with miR-423-5p had a lower level of MALAT-1 compared to the other cell lines (Supplementary Fig. 4A). Then, to test the variability across the samples, principal component analysis (PCA) was performed with log2-transformed raw count data. We observed that the different clones clustered according to the type of modification (Supplementary Fig. 4B). In particular, the cell lines that overexpressed miR-423-5p were separated from the ones transfected with MALAT-1 and the empty vector along the first principal component (PC1), suggesting that the major source of variability is the overexpression of miR-423-5p. The three groups were better separated along the second principal component (PC2). Moreover, we performed three pairwise comparisons among all the groups and explored the biological processes (BPs) enriched by the up and down-regulated genes identified for each comparison. BPs involved in cell movement, proliferation, and epithelial-mesenchymal transition (EMT) were identified as significantly activated (*p*-value $< 0.05$) as shown in the circos plot in Fig. 5 and Supplementary Table 1. Concerning the BPs related to EMT, we observed that most of the genes were down-regulated in cell lines overexpressing MALAT-1 and up-regulated in cell lines overexpressing miR-423-5p. Among them, we found WNT5A, SPRED2, TGFBR2, and EGFR. Correlation between the down-regulation of these genes and the increased capability of HCC cells to migrate and invade has been previously reported [26–29]. We also observed that the MALAT-1+ cell lines overexpress metalloproteinases-15 and −28 (MMP15 and MMP28) that are instead down-regulated in miR-423-5p+ cell lines. The MMPs are proteinases with the capability to reshape the extracellular matrix and favour cell invasion, tumor growth, and angiogenesis. Interestingly, BPs related to autophagy and mitochondria were enriched by the up-regulated genes identified by comparing overexpressing MALAT-1 cell lines to overexpressing miR-423-5p cell lines as shown in Fig. 5D.

### Mitochondrial involvement

**Fig. 6:**
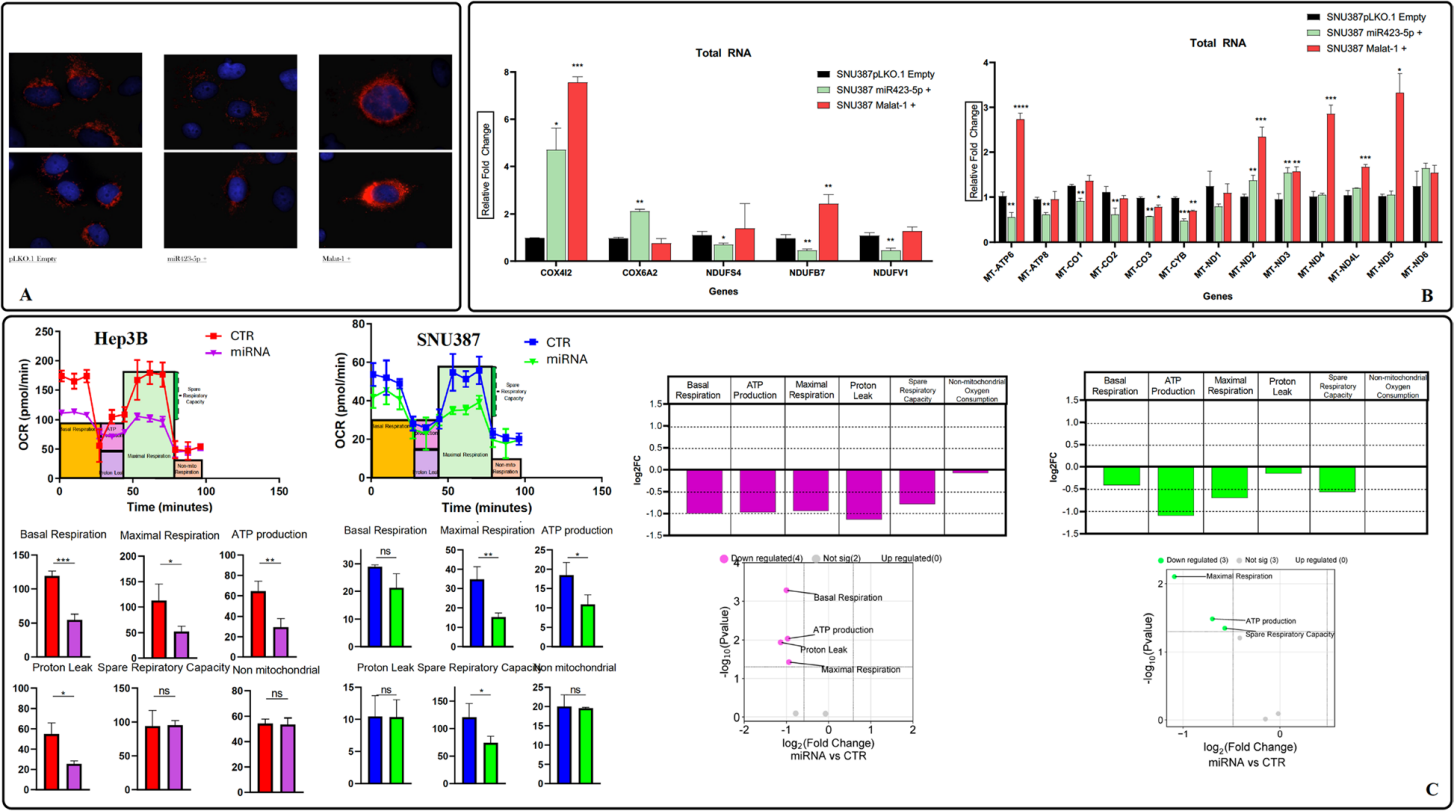
**A** Representative fluorescence microscopy images of mitochondria in SNU387 clones expressing either miR-423-5p or MALAT-1, transfected with MTS-mCherry-GFP1-10 plasmid. miR-423-5p-overexpressing cells showed fewer and smaller mitochondria with a rounded morphology, whereas MALAT-1-overexpressing cells displayed increased mitochondrial number and size. **B** qRT-PCR analysis of mitochondrial-related gene expression in SNU387 clones. Genes encoded by both mitochondrial and nuclear DNA involved in mitochondrial respiration and activity were significantly downregulated in miR-423-5p-overexpressing cells and upregulated in MALAT-1-overexpressing clones. **C** Seahorse Mito Stress Test performed in miR-423-5p-overexpressing SNU387 and Hep3B cells revealed reduced mitochondrial function, including decreased basal respiration, maximal respiration, ATP production, proton leak, and spare respiratory capacity, compared to control cells. * $p < 0.05$, ** *p*-value $< 0.01$, *** *p*-value $< 0.001$, **** *p*-value $< 0.0001$.

The morphological and pathway modifications observed so far with both live cell imaging and whole transcriptome, respectively, led us to hypothesize a metabolic shift involving mitochondria in the miR-423-5p/MALAT-1 transduced cells. In order to study mitochondria number and shape changes, we generated a cell model with easily detectable mitochondria. This was achieved by the use of the MTS-mCherry-GFP1-10-Hyg-N1 plasmid, that was transfected in the already modified clones. After the G418 selection, pure SNU387 miR-423-5p+ and MALAT-1+ clones expressing red fluorescent mitochondria were obtained and screened with fluorescence Leica DM 6000. The panel presented in Fig. 6A demonstrated the different mitochondria number and shape between the distinct clones. In particular, miR-423-5p transduced SNU387 showed less and small-rounded mitochondria if compared to the control, while MALAT-1 SNU387 expressed a higher number of active mitochondria. After these observations, a qRT-PCR primer panel was designed based on the determination of mitochondrial genes encoded by either mitochondrial or nuclear DNA and involved in the mitochondrial activity (as described by Fig. 6B). The histograms showed an overall downregulation of mitochondrial related genes in miR-423-5p overexpressing SNU387, while the MALAT-1+ clone showed a higher expression. This clearly demonstrated the mitochondrial involvement in the miR-423-5p and MALAT-1-induced biological effects in HCC cell models. The same experiment was also performed on MALAT-1+ overexpressing Hep3B cells with similar results (data not shown). To go deeper into mitochondrial function and activity, we performed the MitoStressTest on the Agilent Seahorse platform on both miR-423-5p+ Hep3B and SNU387. We found that Basal respiration, Maximal Respiration, ATP production, Proton leak and Spare Respiratory Capacity were significantly impaired in both models overexpressing miR-423-5p if compared to controls (Fig. 6C), confirming our hypothesis. Finally, to further investigate whether miR-423-5p could directly target mitochondria-related transcripts, we performed an in silico prediction using the TargetScan tool. This analysis revealed several potential targets of miR-423-5p among genes involved in mitochondrial structure and respiration, including NDUFB7, COX4I2, MT-ND4L, and NDUFB4 (Supplementary Table 3). Notably, NDUFB7, a key subunit of Complex I in the electron transport chain, showed a high-confidence 8mer site with a Context++ score of −0.99 (99th percentile) and was experimentally confirmed to be downregulated in our model. These findings suggest that the observed metabolic phenotype may result from both direct and MALAT-1-mediated effects of miR-423-5p on mitochondrial regulation.

### miR-423-5p induces tumor inhibition in HCC mouse orthotopic model

**Fig. 7:**
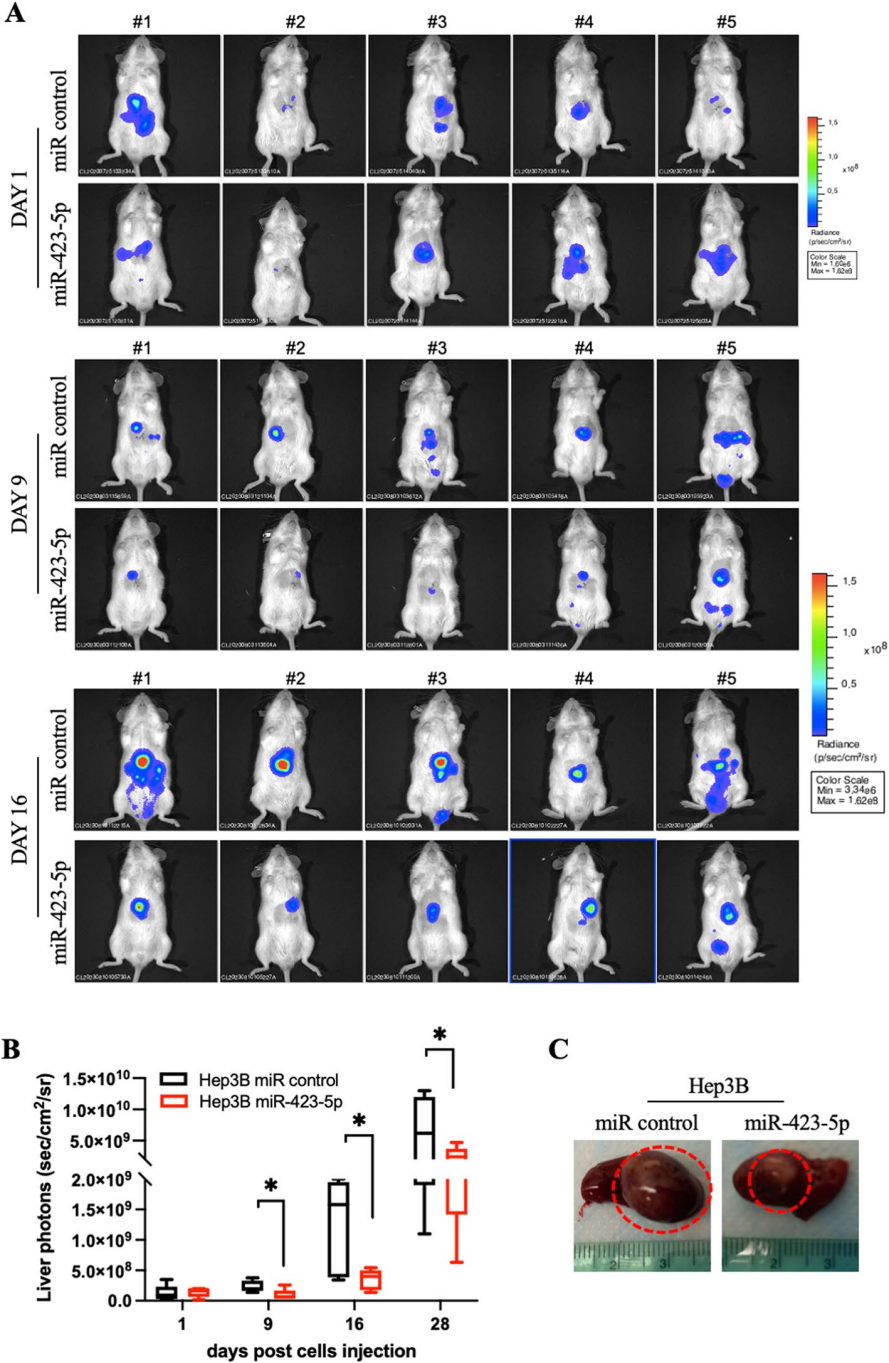
Figure 7. **A** Hep3B-LUC control and miR-423-5p overexpressing cells were injected in the liver of NOD SCID mice. Tumor growth was monitored using the IVIS imaging system 200 series (PerkinElmer). Representative images of mice analysed at days of cells injection (Day 1) and a different times (9, 16). Data were acquired and analysed using the Living Image Software version 4.3. **B** Quantitative analyses of bioluminescence signals in the liver were shown. Luminescent signals are expressed as mean of total flux of photons/sec/cm2/steradian (p/s/cm2/sr). Error bars represent SD. *p*-values were calculated using an unpaired two-tailed t-test; *$p<0.05$. **C** Representative images ex-vivo of Hep3B-LUC control and miR-423-5p tumors in the liver. * $p < 0.05$

We started by investigating the tumorigenic potential of our HCC models. Therefore, we planned a preliminary in vivo experiment in order to set up the experimental conditions for in vivo tumorigenesis of SNU387 cell line. Initially, 1 and 5 million of miR-423-5p+ SNU387 and control cells, respectively, were orthotopically injected in three different animals per group, but unfortunately, the tumor did not growth at any cell density. Therefore, we focused on the Hep3B cells for in vivo orthotopic models (Fig. 7A). Hep3B tumorigenic potential was previously well established and on these bases we implanted luminescent Hep3B cells (LUC), overexpressing miR-423-5p and control, in the liver of NOD SCID mice. Tumor growth was monitored by IVIS imaging. In accordance with the in vitro results, the cells overexpressing miR-423-5p did not generate tumors so efficiently as the control cells and we observed a tumor growth inhibition of about 75% (at day 16 after cells injection) over control tumors (Fig. 7A-B). Control cells generated masses showing a remarkably higher volume and the presence of necrotic and hemorrhagic areas limiting the luciferin uptake of the cells. This caused, at day 28, that the LUC assessment gave a less striking difference between CTR and miR-423-5p generated tumors, if compared to just the caliper measurement of ex-vivo tumors as shown in Fig. 7C. These data demonstrated, also in preclinical animal models, the tumor suppressive role of miR-423-5p in HCC tumors.

### MALAT-1 knockdown confirms the lncRNA role in HCC progression in vivo

**Fig. 8:**
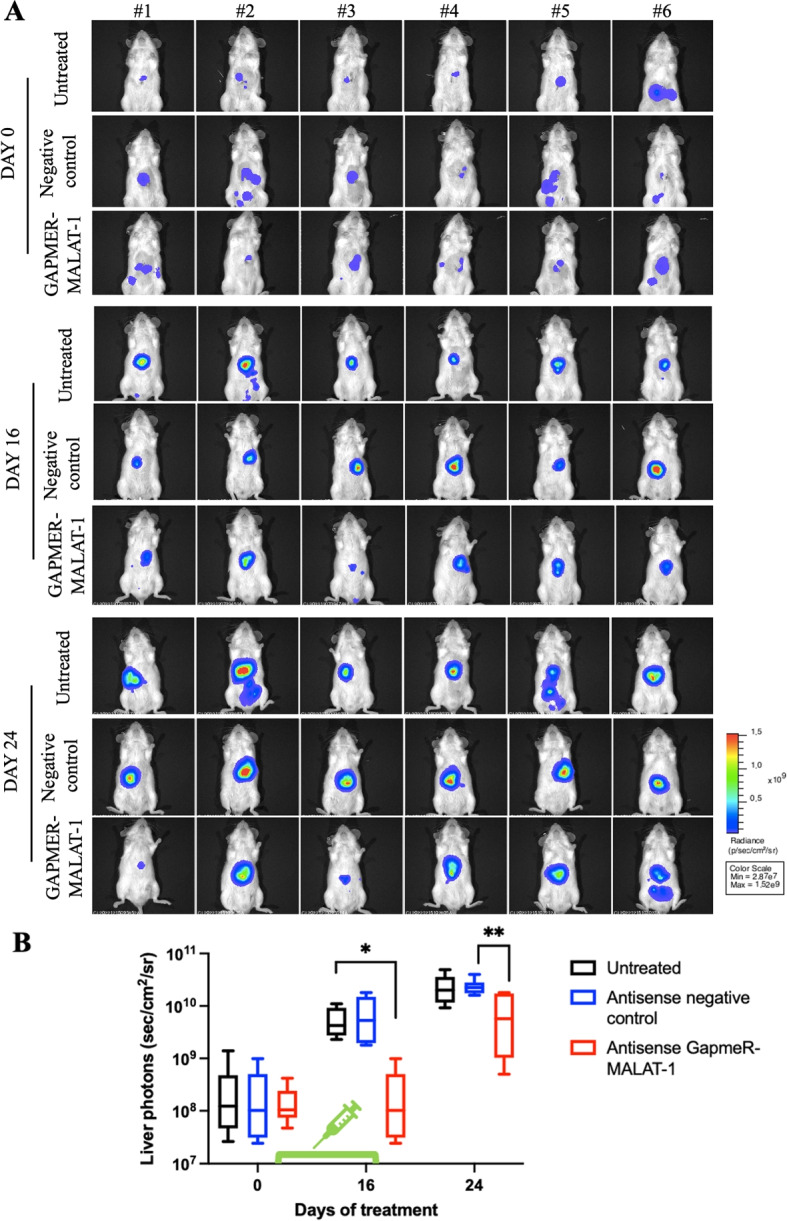
Figure 8**A** Hep3B-LUC cells were injected in the liver of NOD SCID mice. Mice were treated intraperitoneally (ip) with antisense LNA negative control at 25 mg/Kg twice a week for five treatments and with antisense LNA GapmeR-MALAT-1 at 25 mg/Kg twice a week for five treatment. Real-time tumor growth was monitored using the IVIS imaging system 200 series (PerkinElmer). Representative images of mice analysed before starting the treatment (day 0) and at different times (days 16, 24) were shown. Data were acquired and analysed using the Living Image Software version 4.3. **B** Quantitative analyses of bioluminescence signals in the liver were shown. Luminescent signals are expressed as mean of total flux of photons/sec/cm2/steradian (p/s/cm2/sr). Error bars represent SD. *p*-values were calculated using an unpaired two-tailed t-test; * $p < 0.05$, ** *p*-value $< 0.01$

miR-423-5p is involved in several intracellular modulations and is able to interfere with oncogenic processes in HCC. In particular, the miR-423-5p and MALAT-1 inverse correlation was also studied in vivo to strengthen the translational value of miR-423-5p administration and consequent MALAT-1 down regulation. To achieve MALAT-1 knockdown in vivo we used a specific short DNA antisense oligonucleotide structure with RNA-like segments (GapmeR) capable of knocking down MALAT-1 in a very similar way as miR-423-5p does. The results we obtained were encouraging since downregulating MALAT-1 had a significant impact on orthotropic HCC in vivo growth as shown in Fig. 8. This is an exciting starting point to consolidate the translational value of miR-423-5p, since this microRNA is able to do a lot more than just dowregulating the long non-coding RNA MALAT-1, and this is already a striking and extremely interesting capability. In particular, NOD SCID mice treated with GapmeR showed a significative reduction of the tumor in the liver, that reached an inhibition of about 70 % starting from the day 16, and, more interestingly, the stabilization of tumor growth was maintained for approximately 10 days following the end of the treatment. These results demonstrate the efficacy of the inhibition of MALAT-1 in impairing tumor growth additionally supporting the existence of an inverse relationship between the two non-coding RNAs also in vivo. We are currently working on an innovative strategy to deliver miR-423-5p, ensuring both its stability in the bloodstream and concrete uptake in the liver, expecially in the tumor.

## Methods

### Cell lines and growth conditions

We obtained HepG2 and SNU387 HCC cell lines from the American Type Culture Collection (ATCC, Manassas, VA, USA). The Hep3B cell line was obtained from DSZM (Leibniz Institute, Berlin, Germany). These cell lines were cultivated in specific media: Eagle’s Minimum Essential Medium for HepG2 and Hep3B (BE12-6621, Lonza, Basel, Switzerland), and RPMI 1640 Medium for SNU387 (12-167Q, Lonza, Basel, Switzerland). To the media, we added 10% (v/v) fetal calf serum and 1 (w/v) L-glutamine (Lonza). The cells were incubated at 37 °C in an environment with 5% (v/v) CO2 and 100% (v/v) humidity. For the generation and culture of stable transfected cell lines expressing Mimic hsa-miR-423-5p, we employed the same culture media with the addition of 1.5 $\mu$g/mL of Puromycin for Hep3B and SNU387, and 2.5 $\mu$g/mL of Puromycin for HepG2 to ensure antibiotic selection. As for the HEK293T cell lines, these were acquired from the American Type Culture Collection (Manassas, VA, USA). They were maintained in DMEM media (BE12-604F, Lonza, Basel, Switzerland), supplemented with 10% (v/v) fetal calf serum and 1% (w/v) L-glutamine (Lonza).

### Transient transfection of HCC cells with miRNA 423-5p mimic and inhibitor

To clarify the specific role of miRNA 423-5p, HCC cells at 80% of confluence were transfected with miRNA 423-5p mimic and its inhibitor. The day before transfection, cells were trypsinized and seeded in an appropriate medium without antibiotics in 12-well plates. MiRIDIAN miR-423-5p mimic, miRIDIAN miR-423-5p hairpin inhibitor, and their controls with unrelated sequences (Dharmacon, Lafayette, CO) were transfected at 50 nmol/l. Transfections were performed by using Lipofectamine 2000 (Invitrogen, Carlsbad, CA), as described by the manufacturer. After 6 hours, the transfection mix was replaced with complete medium. The analyses were performed 72 hours after transfection.

### Generation of mimic miR-423-5p HCC cell lines using lentiviral transduction system

HCC cell lines HepG2, Hep3B and SNU387 were seeded in a 96 multiwell plate and the following day infected with empty vector (MISSION® pLKO.1-puro Empty Vector Control Plasmid DNA; Sigma-Aldrich) or miRIDIAN microRNA hsa-miR-423-5p shMIMIC lentiviral particles (Lentiviral Human TurboGFP shMIMIC; Thermo Scientific) at a multiplicity of infection (MOI) between 2.5 and 20. At 48 h post-infection, GFP fluorescent cells were detected by fluorescent microscopy and Puromycin (Invitrogen) was added to the medium (1.5 $\mu$g/ml and 2.5 $\mu$g/ml - established by killing curve assay performed on the wild type cells). The medium was changed every 2 days, and puromycin-resistant cells were selected and expanded. Successful transduction was assessed by positive tGFP expression, and miR-423-5p expression was confirmed by analyzing mature miR-423-5p expression using RT-qPCR. Hep3B LUC cells were obtained with a stable infection with lentiviral luciferase vector pRRLSIN.cPPTLuciferase (Addgene, Watertown, Massachusetts, USA).

### Bacteria transformation and plasmids amplification

The pMD2.G, VSVG (Envelope), psPAX2 (Packaging), pLKO.1-puro Empty plasmids were amplified individually using XL1-Blue super-competent bacteria. First, the cells were thawed on ice for 5 minutes and mixed before transferring 50 $\mu$L to a chilled sterile polypropylene culture tube. 100 μg of each plasmid were added to the competent cells and placed on ice for 10 minutes. Cells were heat-shocked at 42 °C for 45 seconds, placed on ice for 2 minutes, added to 450 $\mu$L of lysogeny broth (LB; Sigma-Aldrich) containing 100$\mu$g/mL ampicillin (Sigma-Aldrich) and were shaken at 37 C° for 60 minutes. All 500 $\mu$L aliquot was plated on ampicillin-containing LB-Agar plates overnight, and afterwards, clones were picked and shaken overnight at 37 °C in 5 mL of LB broth containing ampicillin. The plasmid was extracted using the QIAprep® Miniprep (QIAGEN). Cells were pelleted in a centrifuge and resuspended in 250 $\mu$L of Buffer P1. 250 $\mu$L of Buffer P2 was added and mixed by inverting the tube. Then 350 $\mu$L of neutralization solution (N3) was added and mixed by inverting the tube. Cells were centrifuged for 10 minutes at 14,000 x g. The supernatant was transferred to a QIAprep 2.0 spin column and centrifuged for 1 minute at 12,000 x g. The flow-through was discarded, and two washes of 500 $\mu$L wash solution were performed, and the column was centrifuged for 1 minute at 12,000 x g each time. The plasmids were eluted from the column by adding 50 $\mu$L of elution buffer, waiting 2 minutes and centrifuging the columns at 12,000 g for 2 minutes. The concentration of the plasmids were then measured using a NanoDrop ND1000.

### Lentiviral particles production

Target sequence plasmid, together with appropriate packaging plasmid and envelope plasmid, were transfected in HEK293T (Passage 3 maximum) using lipofectamine 2000. After 24 hours from transfection, HEK293T media containing Lentiviral Particles Fraction 1 was collected, filtered with a 0.22 μM syringe filter and split evenly in 1 mL cryovials for storage at −80°C. Fresh media was added to cells. After 48 hours from transfection, HEK293T media containing Lentiviral Particles Fraction 2 was collected, filtered with a 0.22 μM syringe filter, and split evenly into 1 mL cryovials for storage at −80°C. 1 mL of LVPs was able to transduce efficiently 80% of Wild Type cells (measured by GFP fluorescence) seeded in 6 multiwell plate.

### Generation of MALAT-1 HCC cell lines using a lentiviral transduction system

HCC cell lines Hep3B and SNU387 were seeded in 6 multiwell plates and treated with 1 mL MALAT-1 lentiviral particles after 24 hours (HEK293T cell product, LVP titer not assessed). At 48 h post-infection, GFP fluorescent cells were detected by fluorescent microscopy and Puromycin (Invitrogen) was added to the medium (1.5 $\mu$g/ml established by Killing curve assay). The medium was changed every 2 days, and puromycin-resistant cells were selected and expanded. Successful transduction was assessed by positive tGFP expression, and MALAT-1 expression was confirmed by analysis of the long non-coding RNA expression using RT-qPCR.

### RNA immunoprecipitation

The RNA immunoprecipitation (RIP) assay was performed using extract obtained from SNU387 clones with Magna RIP RNA-binding protein immunoprecipitation kit (MilliporeSigma) according to manufacturer protocol. Briefly, the cells were scraper detached and lysed, the magnetic beads were prepared using AGO2 RIP antibody (MilliporeSigma) and a negative control IgG antibody. Immunoprecipitation was conducted overnight at 4 °C, then RNA purified with phenol-chloroform-isoamyl alcohol. FOS primers were used to check AGO2 enrichment and successful specific immunoprecipitation by qPCR while target RNAs related cDNA and expression analysis by qPCR was performed according to the methods described below.

### CyQuant proliferation assay

Cells were detached from respective plates and seeded in a 96-well plate at a density of 1500 per well in sextuplicate for the CyQUANT NF Cell Proliferation Assay (C35006, Thermo Fisher Scientific). At 24 and 48 hours after seeding, cells were treated with the CyQUANT reagent for 30 minutes. The fluorescence readout was measured using the plate reader Infinite M200 Pro TECAN (Tecan Group Ltd, Männedorf, Switzerland) at 485 nm excitation and 530 nm emission. The CyQUANT NF Assay is based on the measurement of cellular DNA content via fluorescent dye binding. Because cellular DNA content is highly regulated within a specific cell population, it is closely proportional to cell number. All proliferation experiments were performed at least three times independently.

### Proliferation assay – live-time imaging with IncuCyte

Cells were detached from respective plates and seeded in a 96-well plate at a density of 2500 and 5000 per well in sextuplicate for each cell concentration. Plates were then stored into IncuCyte Live-Time microscope incubator (37°C, 5% CO2) for a maximum of 120 hours. Capture protocol was set for 3 different captures per well, once every 30 minutes. The confluence percentage was recorded and plotted by IncuCyte software considering all the captures for each time point.

### Cell migration and invasion assays

For wound healing assay (Scratch assay), HCC cells were cultured to 80% confluence and serum-starved for 24 h in 24 multiwell plate, then vertical scratches were drawn through the confluent monolayer of cells using a 10-$\mu$l pipette tip. After being washed with PBS to remove any cell debris caused by induction of the wound, images from triplicate experiments at 0 h, 12 h, 24 h were taken for Hep3B, and at 0 h, 24 h, 48 h were taken for HepG2 and SNU387. The distances between the scratch edges were measured at three different points using Carl Zeiss AxioVision software (Carl Zeiss, Oberkochen, Germany). The measurements were expressed as percentages of the wound area. Independent experiments were performed three times, with three separate wells per condition. For the chamber-based cell migration and invasion assay Cultrex 96-Well BME Cell Invasion Assay (3455-096-K, Cultrex) was used following the manufacturer’s recommendations (Trevigen, Gaithersburg, MD, USA). These assays employ a simplified Boyden chamber design with an 8-micron polyethylene terephthalate (PET) membrane. Briefly, the top chamber was coated with 0.5 X BME solution at 37 °C in a CO2 incubator for 4 hours and then added cells suspension in serum-free medium. The bottom chamber was filled with FBS containing medium and incubated at 37 °C with 5% CO2 for 48 hours. After washing the cells in the bottom chamber, they were labelled by Calcein AM (Trevigen), and the measure of the number of cells invaded or migrated was performed using the plate reader Infinite M200 Pro TECAN (Tecan Group Ltd, Männedorf, Switzerland) at 485 nm excitation and 520 nm emission. All experiments were independently repeated at least three times.

### Colony formation assay

HCC cell lines were seeded in 6 MW at low density (2.5 × 10^5^ cells/well) in culture media with low percentage FBS (1%). After 14 days, cells were stained with Crystal Violet, and the colonies were evidenced. The number and size of self-generated colonies were recorded and compared with the controls.

### RNA extraction

Total RNA was extracted using the QIAGEN miRNeasy mini kit (QIAGEN, Cat-74104) or NORGEN Total RNA Purification Kit (Cat. 17200, 37500, 17250) following the manufacturer’s instructions. Briefly, pellet cells were lysed directly adding 250 $\mu$L Buffer RTL + 2 $\mu$L beta-mercaptoethanol. Then, 1 volume of 70% ethanol was added to the lysate, mixed well and transferred in a Mini spin column for centrifuge at 8000 x g for 15 sec. The columns were washed once with 350 $\mu$l buffer RW1 and twice with 500 $\mu$l buffer RPE with centrifugation at 8000 g for 1 min at room temperature between each wash. The columns were placed into a fresh collection tube and centrifuged at 14000 g for 2 min at room temperature to remove any buffer contaminants. Spin columns were then transferred to new RNase-free tubes and RNA was eluted by adding 50 $\mu$l RNase-free H2O to the columns and centrifuging at 8000 g for 1 min at room temperature. RNA was stored at −20°C for a short time. Total RNA was quantified using the NanoDrop 8000 Spectrophotometer (Thermo Scientific).

### miRNA reverse transcription for miRNA expression analysis

For miRNA expression detection, cDNA was synthesized using stem-loop reverse transcription primers using the TaqManTM microRNA reverse transcription kit (Applied Biosystems, Paisley, UK) according to the manufacturer’s instructions. Each reaction contained 5 $\mu$l of 2 ng/$\mu$l sample RNA, 0.15 $\mu$L of 100mM dNTPs, 1$\mu$L 50U/$\mu$l MultiScribe Reverse Transcriptase, 0,19 $\mu$L 20U/$\mu$l RNase Inhibitor, 1,5 $\mu$L 10 X Reverse Transcriptase buffer, 4.16 $\mu$l RNase-free water and 3$\mu$L Taqman® miRNA Reverse Transcription Primers 5X for the mature sequence of the miRNA of interest. Endogenous control RNU6 was also performed for each sample to normalize changes in the miRNA expression. The mature sequence of miRNA of interest and endogenous control sequences are: - hsa-miR-423-5p 5’ UGAGGGGCAGAGAGCGAGACUUU 3’ - U6snRNA 5’GTGCTCGCTTCGGCAGCACATATACTAAAATTGGAACGATACAGAGAAGATTAGCATGGCCCCTGCGCAAGGATGACACGCAAATTCGTGAAGCGTTCCATATTTT 3’. cDNA synthesis reactions were incubated at 16 ºC for 30 min, 42 ºC for 30 min, and 85 ºC for 5 min and then held at 4 ºC. Samples were stored at −20 ºC before quantitative PCR was performed.

### mRNA reverse transcription for gene expression analysis

For first-strand cDNA synthesis from RNA was prepared following the manufacturer’s instructions described below. Into a 0.5ml Eppendorf tube, 2 $\mu$g of RNA, 1 $\mu$l Oligo (DT)15 primer (Promega) and ddH2O were added to achieve a final volume of 10 $\mu$l. The samples were then heated using UNO-Thermoblock to 70 °C for 5 minutes to melt secondary structures. Samples were then immediately put on ice for 5 minutes before the following were added to each sample: 5 $\mu$L M-MLV 5X Reaction Buffer (Promega), 1$\mu$L M-MLV Reverse Transcriptase (Promega), 0.7$\mu$L RNasin Ribonuclease Inhibitor (Promega), 1$\mu$L Deoxynucleotide Set (dNTPS) (Sigma) and 7.3 $\mu$L Nuclease Free water. Samples were mixed gently and incubated for 60 minutes at 40 °C in a water bath. After incubation, samples were finally heated using an UNO-Thermoblock to 95 °C for 5 minutes and immediately stored at −20°C, ready for qRT-PCR.

### TaqMan quantitative real-time PCR

miRNA qRT-PCR was performed using Taqman® miRNA Assay RT-PCR Probes and TaqMan® Universal Mastermix PCR (containing AmpliTaq Gold® DNA polymerase, dNTP mixture and optimal salt conditions, Invitrogen) on Rotor-Gene PCR cycler (Qiagen) in accordance with the manufacturer’s instructions. Each reaction master mix contained 250 nM TaqMan probe, 1 X Taqman Universal PCR MasterMix II (Invitrogen) and nuclease-free H2O to a total volume of 18,67 $\mu$l per sample. cDNA was pipetted in technical triplicate, 1.33 $\mu$l per well, and the master mix was added to each well to give a total reaction volume of 20 $\mu$l. RNU6 was used as an endogenous control in separate wells. Thermal cycling as follows: 2 min incubation at 50ºC, 10 min incubation at 95 ºC then 40 cycles of 15 sec at 95 ºC and then 60 sec at 60 ºC.

### Quantitative qRT-PCR for mRNA expression

qRT-PCR was set up into PCR tubes using Sybr Green SuperMix on Rotor-Gene PCR cycler (Qiagen) following manufacturer’s instructions described here per sample: 6.75 $\mu$L iTaq™ Universal SYBR® Green Supermix (Bio-Rad), 0.5 $\mu$L 10 pM Forward Primer (MALAT-1 5’ GGATCCTAGACCAGCATGCC 3’; GAPDH 5’ ACCCACTCCTCCACCTTTGA 3’ HPRT1 5’ CATTATGCTGAGGATTTGGAAAGG 3’), 0.5 $\mu$L 10 pM Reverse Primer (MALAT-1 5’AAAGGTTACCATAAGTAAGTTCCAGAAAA 3’; GAPDH 5’ CTGTTGCTGTAGCCAAATTCGT 3’ HPRT1 5’ CTTGAGCACACAGAGGGCTACA 3’) and 2.25$\mu$L Nuclease Free water for a total volume of 10 $\mu$L. 2 $\mu$L of 50 ng cDNA was pipetted in technical triplicate per well and the master mix added to each well to give a total reaction volume of 12 $\mu$l. GAPDH was used as an endogenous control in separate wells. In cases of Non-Template Controls (NTC), cDNA template was substituted with ddH2O. Thermal cycling conditions began with 5 min incubation at 95 °C, then followed by 40 cycles of 10 sec at 95 ºC, 20 sec at 60 ºC and then 20 sec at 72 ºC. The primers used are design and product by Eurofins Genomics. Additional Melt analysis to assess the quality of the experiment is performed at the end of the run. Mitochondrial related transcripts primers are reported in Supplementary Table 2. Results were shown relative to the control sample using the ${\Delta }\Delta$Ct method and expressed as relative fold change. For miRNA expression, results were normalised to U6. For mRNA and long non-coding RNA expression, results were normalised to GAPDH or HPRT1.

### Fluorescent mitochondria cell lines generation

HCC cell lines were transfected with MTS-mCherry-GFP1-10-Hyg-N1 (Addgene plasmid # 91957) using Lipofectamine 2000 according to the manufacturer protocol. Pure population was then selected using G418 antibiotic (Sigma-Aldrich). Mitochondria were visible (Red) in vital cells using fluorescent microscopy (LEICA DM 6000 B 100x and 60x Oil), and the number and shape were recorded and compared between the cell lines.

### Transcriptome analysis

RNA was extracted from HCC cell clones using Total RNA Purification Kit (Norgen Biotek Corp) according to the manufacturer protocol. Each cell clone was cultured in three separate flasks to achieve biological triplicates. RNA quantification and quality assessment were performed using NanoDrop 1000 Spectrophotometer (Thermo Fisher Scientific), Qubit RNA HS Assay Kit (Thermo Fisher Scientific) and Bioanalyzer Total RNA Pico Chip (Agilent). SuperScript VILO cDNA Synthesis Kit (Thermo Fisher Scientific) was used to obtain cDNA according to manufacturer protocol. IonChef (Thermo Fisher Scientific), related Ion AmpliSeq Kit for Chef DL8 (Thermo Fisher Scientific) and Trascriptomic panel (Thermo Fisher Scientific) were used to obtain sequencing libraries and sequencing chip template. The NGS runs were performed on IonS5 (Thermo Fisher Scientific) on Ion 540 Chip (Thermo Fisher Scientific).

### In vivo experiment

All animal procedures were in compliance with the national and international directives (D.L. March 4, 2014, no. 26; directive 2010/63/EU of the European Parliament and European Council; Guide for the Care and Use of Laboratory Animals, U.S. National Research Council, 2011;Animal Research guidelines Reporting of In Vivo Experiments (ARRIVE) guidelines) and approved by the Italian Ministry of Health (authorization n.346/2020-PR, released on April 2020). Mice were maintained in a barrier facility on high-efficiency particulate air HEPA-filtered racks and received food and water ad libitum. NOD SCID (6 weeks old) male immunodeficient mice (Charles River Laboratories, Calco, Italy) were orthotopically injected in the liver with 1$\times$10^6^ of Hep3B control or miR-423-5p-LUC over expressing cells using an insulin syringe with a 27-gauge needle. Mice were anesthetized with a combination of tiletamine–zolazepam (Telazol, Virbac, Carros, France) and xylazine (xylazine/Rompun, Bayer, Leverkusen, North Rhine-Westphalia, Germany) given intramuscularly at 2 mg/kg. Following the anesthesia, a parallel incision to the linea alba is made in the abdominal wall to expose the liver. Then, 10 μl of Hep3B cells are mixed with 10 μl of Matrigel and injected in the liver very slowly. After injection, the peritoneum was sealed using absorbable suture (PolySorbTM 6-0) and the skin layer of the abdominal wall was close by absorbable suture (PolySorbTM 5-0). Finally, mice were medicated with an oral administration of 0.5 mg/Kg of Metacam (meloxicam) to control post-operative pain and inflammation. Real time tumor growth was monitored using the IVIS Lumina II CCD camera system (PerkinElmer, Shelton, Connecticut, USA) by intraperitoneal injection with 150 mg/Kg D-Luciferin (PerkinElmer). Bioluminescence signals were determined by the number of photons and were acquired and analyzed using the Living image software version 4.3 (PerkinElmer). Each experimental group included 5 mice. For the antisense GapmeR-MALAT-1 experiment, NOD SCID (6 weeks old) male immunodeficient mice were orthotopically injected in the liver with 1$\times$10^6^ Hep3B-LUC cells as described above. Seven days after cell injection, mice were randomized and treated intraperitoneally (ip) with antisense LNA negative control (Qiagen n.3545711) at 25 mg/Kg twice a week for five total treatments, with antisense LNA GapmeR-MALAT-1 (Qiagen n.3545711) at 25 mg/Kg twice a week. Real time tumor growth was monitored weekly using the IVIS Lumina II CCD camera system (PerkinElmer, Shelton, Connecticut, USA) by intraperitoneal injection with 150 mg/Kg D-Luciferin (PerkinElmer). Bioluminescence signals were determined by the number of photons and were acquired and analyzed using the Living image software version 4.3 (PerkinElmer). Each experimental group included 6 mice. The student’s t-test (unpaired, two-tailed) was used for single pair-wise comparisons. Differences were considered statistically significant when $P < 0.05$. The student’s t-test (unpaired, two-tailed) was used for single pair-wise comparisons. Differences were considered statistically significant when $P < 0.05$.

### Statistical and bioinformatic analysis

All data are presented as the mean ± SEM for at least three independent experiments. For each experiment, the statistical tests are indicated in the results section. The student’s t-test was conducted using Prism 8 (Graphpad Software, La Jolla, CA, USA) to compare the means. Significant (‘*’/$p= < 0.05$), very significant (‘**’/$p= < 0.01$), highly significant (‘***’/$p= < 0.001$) or very highly significant (‘****’/$p= < 0.0001$). Other parametrical and non-parametrical analyses were carried out using IBM SPSS Ver. 25, *p*-value $<0.05$ is considered statistically significant. Survival analysis and Kaplan-Meier curves for Overall Survival (OS) and Disease-Free Survival (DFS) were generated using the Kaplan-Meier Plotter online tool (KM Plotter, http://kmplot.com/analysis), which integrates publicly available transcriptomic datasets from liver cancer patients. Differential expression analyses were performed using DESeq2 v1.36.0 R package (1). Before performing the differential expression analyses the genes with 10 counts in at least two samples were selected. Genes with an absolute value of log2 fold-change (FC) greater than 1 and adjusted *p*-value less than 0.01 were selected as differentially expressed for each comparison. The up- and down-regulated genes selected for each comparison were used to perform a gene set enrichment analysis with DAVID web server (2, 3). Biological processes with Fisher-exact *p*-value less than 0.05 were selected for downstream analyses. The circos plots were made using the circlize R package (4). The analyses were performed using R Statistical Software v4.2.1.

## Discussion

Several studies reported a role for both miR-423-5p and MALAT-1 in the regulation of crucial tumor processes including proliferation, migration and cell metabolism. In detail, miR-423-5p was described in different cancer contexts as a potentially oncosuppressor microRNA, able to inhibit tumor growth and invasion. In ovarian cancer, miR-423-5p overexpression caused a significant reduction in the functional capabilities of tumor cells, impairing proliferation, invasion and colony formation [24]. Similar results were observed in colon cancer, where miR-423-5p was found to be involved in apoptosis and oxidative stress response [12]. In our previous studies on prostate cancer, this microRNA showed a very interesting and promising capability to reduce the progression and the aggressiveness of PCa both in in vitro and in vivo models [13]. In the latter PCa model, we have demonstrated that miR-423-5p binds the lncRNA MALAT-1 reducing its expression and inducing metabolic reprogramming of PCa cells [25]. Similar roles have been observed in other cancers: Wan et al. showed that miR-423-5p reduced glycolysis and metastatic ability in osteosarcoma through Wnt5a/Ror2 signaling [30], while Wen et al. linked the circRNA-miR-423-5p-SOX4 axis to altered glycolysis and tumor growth in papillary thyroid carcinoma [31]. MALAT-1 is widely studied and its oncogenic role is well recognized as it promotes migration and invasion but can also act on proliferation and programmed cell death pathway avoidance, interfering with specific molecular mechanisms such as the activation of PI3K/AKT and mTOR [18]. MALAT-1 regulates ferroptosis and mitochondrial stability via the NUDT16L1 pathway [32]. It also plays a systemic metabolic role, as shown by ceramide-triggered MALAT-1-rich extracellular vesicles that enhanced mitochondrial function and cell migration [33]. Wang et al. highlighted MALAT-1’s ability to modulate oxidative stress, cell death, and mitochondrial injury in diabetic heart disease through miRNA sponging and RhoA/ROCK signaling [18]. Given that, our specific trigger for this work was the observation by our previous studies suggesting that HCC patients responsive to sorafenib had higher circulating levels of miR-423-5p which was, in turn, correlated with the induction of autophagic cell death in an in vitro model of HCC [20]. miR-423-5p was then correlated in silico with MALAT-1 thanks to the several and strong binding sites predicted, especially in the MALAT-1 regulatory sequence. This is why we first checked patient online datasets and obtained the Kaplan-Meier curves on OS and DFS to have an idea of the clinical impact of these two non-coding RNAs. Indeed, we found that miR-423-5p had a favorable impact on both OS and DFS of HCC patients while MALAT-1 a detrimental effect. Therefore, we decided to investigate on the molecular mechanisms and the specific interaction between miR-423-5p and MALAT-1 in HCC in vitro models. To do this, we transiently and stably overexpressed miR-423-5p and MALAT-1 in HCC cell models. Transient transfections of miR-423-5p mimic and inhibitor into HCC cell lines revealed that the miRNA mimic caused a decreased MALAT-1 expression. On the other hand, miRNA inhibitor induced a significant MALAT-1 increase compared to the control. These promising findings from transient modulations pushed us to establish stable overexpressing models for additional experiments. To do so, we employed the lentiviral infection technology, generating clones stably expressing these two non-coding RNAs. First of all, we checked again MALAT-1 expression in the stable miR-423-5p overexpressing HCC models (HepG2, Hep3B and SNU387). Also in this case, the inverse correlation between miR-423-5p and MALAT-1 was confirmed: MALAT-1 expression was significantly reduced in miR-423-5p overexpressing clones and viceversa. The inhibitor of miR-423-5p transfected in the miR-423-5p overexpressing cells was able to revert the phenotype as a proof of concept. Then, we aimed to confirm also the physical interaction among these two non-coding RNAs using two different methods: dual-luciferase and RIP assay. Both demonstrated that miR-423-5p and MALAT-1 were interacting in our HCC models. Interestingly, RIP assay demonstrated the physical interaction of the two non coding RNAs in the Ago-2 complex immediately before the interaction with their putative intracellular targets. The subsequent functional studies we conducted were showing a consistent modulation of proliferation, migration, invasion, and clonogenic potential: miR-423-5p overexpression induced a milder, less aggressive cancer phenotype, significantly reducing the aforementioned biological effects in all generated HCC cell models compared to their respective controls due, at least in part, to the downregulation of the lncRNA MALAT-1 that, in turn, caused an opposite effect, above all on migration and invasion. To gain a deeper understanding on what we observed, we performed a wide range of discovery assays that yielded confirmatory results. The whole-transcriptomics sequencing revealed distinct transcript expression patterns between miR-423-5p+ clones and control clones, with the control and MALAT-1+ clones exhibiting a specific aggressive gene signature if compared to the miRNA overexpressing ones. The mitochondrial involvement under this non coding RNA loop after our analyses appeared evident. On these bases, we have investigated upon the effects of the non coding RNA expression on both number and function of mitochondria in our experimental models. By fluorescent microscopy of mitochondria tagged with a specific fluorescently labelled marker, we have found that miR-423-5p significantly reduced the number of mitochondria also affecting their shape that became more rounded. Opposite effects were obtained with MALAT-1 transfection: the number of mitochondria increased showing an elongated functional aspect. By Real Time PCR validation on the mitochondrial related transcripts we found again a significant decrease of almost all transcripts in miR-423-5p overexpressing models and again opposite effects were observed after MALAT-1 stable transfection. Additionally, we have evaluated the impact of these changes on the energetic metabolism with Seahorse MitoStressTest confirming a strong reduction of Basal respiration, Maximal Respiration, ATP production, Proton leak and Spare Respiratory Capacity in two miR-423-5p overexpressing models if compared to controls. Our data suggest double role of miR-423-5p in mitochondrial metabolism regulation. Although MALAT-1 suppression appears to mediate a part of this effect, in silico predictions also identified direct miR-423-5p binding sites in the 3’ UTR of several mitochondrial genes such as NDUFB7, COX4I2, and MT-ND4L. Among them, NDUFB7 was experimentally validated as downregulated in our models, supporting a potential direct interaction. Thus, the regulatory mechanism of miR-423-5p on mitochondrial function in HCC may rely on both direct and indirect pathways. Finally, we evaluated the effects of stable miR-423-5p overexpression in in vivo animal models. To do so, we performed our experiments by the injection of Hep3B controls and miR-423-5p overexpressing cells directly in the liver of immunosuppressed mice (orthotopic models). In Hep3B miR-423-5p overexpressing tumors, we observed a tumor growth inhibition of 75% compared to the controls tumors, demonstrating the antitumorigenic effect of miR-423-5p overexpression. Similar results were observed when we treated mice with GapmeR-MALAT-1 that caused a significant inhibition of tumor growht in the liver. Overall, our results indicated that miR-423-5p in liver cancer has promising translational value, thanks to its capability to interfere with MALAT-1 and its pro-tumorigenic role. Moreover, there are evidence that the overexpression of the microRNA is linked to mitochondrial activity suppression. It is capable of controlling cancerous progression and motility both in vitro and in vivo, pushing us to continue with further studies in order to bring this as a potential treatment. Although our current study focused on functional and mechanistic characterization, we aknowledge that the therapeutic potential of miR-423-5p strongly depends on the development of efficient delivery strategies. Several technologies are currently being explored for RNA-based therapies and our group is actively working on the optimization of stabilized and liver-targeted delivery systems for miR-423-5p, which will represent the next step toward its therapeutic application in HCC.

## Conclusion

In conclusion we showed the tumor suppressive role of miR-423-5p in HCC, and started to understand its complex mechanisms both describing its inverse correlation and interaction with the detrimental long non-coding RNA MALAT-1 and its role in the modulation of mitochondrial function. We employed several in vitro functional studies and also in vivo approaches to confirm our findings. This will be an interesting starting point to disclose the prognostic, diagnostic and even the possible therapeutic role of this interesting microRNA since our group is actively working on an innovative delivery method, capable of preserving the microRNA stability in the bloodstream and ensure the delivery to the tumor.

## Supplementary Information

Supplementary Material 1.

## Data Availability

No datasets were generated or analysed during the current study.
