# Correction: MiR-423-5p is a metabolic and growth tuner in hepatocellular carcinoma via MALAT-1 and mitochondrial interaction

**DOI:** 10.1186/s13046-025-03562-w

**Published:** 2025-11-07

**Authors:** Marco Bocchetti, Alessia Maria Cossu, Manuela Porru, Maria Grazia Ferraro, Carlo Irace, Rossella Tufano, Giovanni Vitale, Gabriella Misso, Nicola Amodio, Marianna Scrima, Ines Simeone, Michele Ceccarelli, Ugo Chianese, Lucia Altucci, Vincenzo Desiderio, Tarik Regad, Michele Caraglia, Silvia Zappavigna

**Affiliations:** 1 Molecular Oncology and Precision Medicine Laboratory, Biogem IRGS, Contrada Camporeale, Ariano Irpino, AV 83031 Italy; 2 Department of Life Sciences, Health and Health Professions, Link Campus University, Via del Casale di San Pio V 44, Rome, RM 00165 Italy; 3 Department of Precision Medicine, University of Campania “Luigi Vanvitelli”, Via De Crecchio, Naples, NA 80131 Italy; 4 John van Geest Cancer Research Centre, Nottingham Trent University, Clifton Lane, Nottingham NG121AS, Clifton, UK; 5 Translational Oncology Research Unit, IRCCS - Regina Elena National Cancer Institute, Via Elio Chianesi 53, Rome, RM 00144 Italy; 6 Department of Molecular Medicine and Medical Biotechnology, University of Naples “Federico II”, Via Pansini, Naples, NA 80131 Italy; 7 Department of Pharmacy, University of Naples “Federico II”, Via Domenico Montesano, Naples, NA 80131 Italy; 8 Laboratory of Computational Biology, Biogem IRGS, Contrada Camporeale, Ariano Irpino, AV 83031 Italy; 9 Department of Electrical Engineering and Information Technologies, University of Naples “Federico II”, Naples, NA Italy; 10 Laboratory of Geriatric and Oncologic Neuroendocrinology Research, IRCCS, Istituto Auxologico Italiano, Milan, MI 20122 Italy; 11 Department of Medical Biotechnology and Translational Medicine, University of Milan, Milan, MI 20122 Italy; 12 Department of Experimental and Clinical Medicine, Magna Graecia University of Catanzaro, Viale Europa, Catanzaro, CZ 88100 Italy; 13 Humanitas Research Hospital, Via Manzoni, Rozzano, MI 20089 Italy; 14 Department of Public Health Sciences, Miller School of Medicine, University of Miami, Miami, FL USA; 15 Department of Experimental Medicine, University of Campania “Luigi Vanvitelli”, Via De Crecchio, Naples, NA 80131 Italy

**Correction: J Exp Clin Cancer Res 44**,** 239 (2025)**

**https://doi.org/10.1186/s13046-025-03524-2**

Following publication of the original article [1], the authors identified an error in Fig. 8 due to an unintentional oversight during figure assembly, the same images were mistakenly used for both the Control and Negative Control conditions at the 16-day time point.

**Incorrect Fig. 8**

**Fig. 8:**
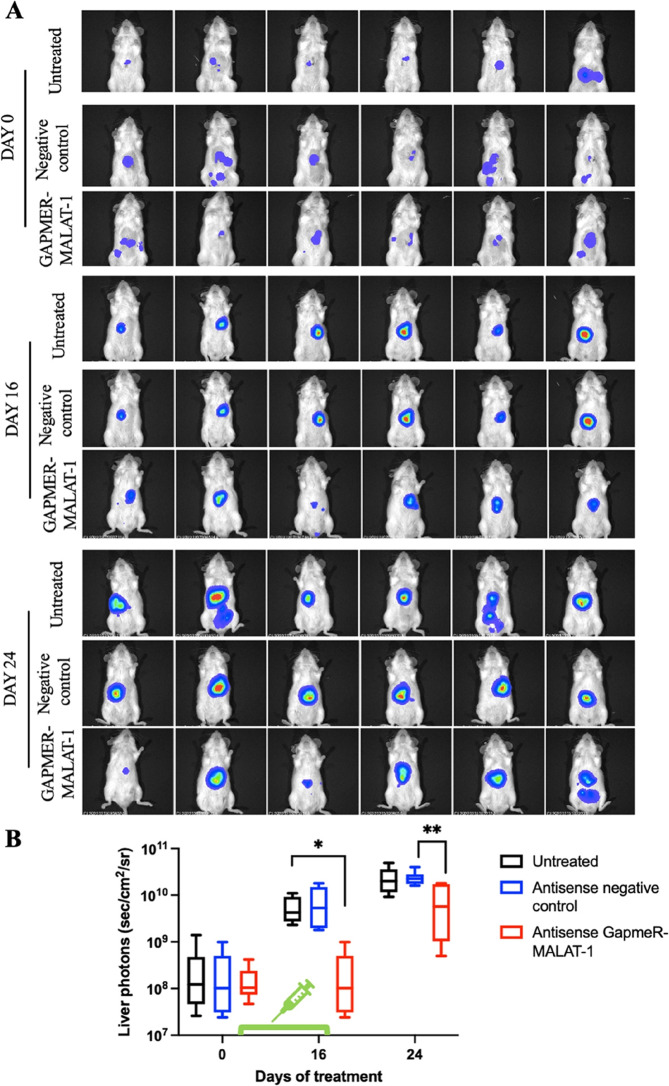
**A** Hep3B-LUC cells were injected in the liver of NOD SCID mice. Mice were treated intraperitoneally (ip) with antisense LNA negative control at 25 mg/Kg twice a week for five treatments and with antisense LNA GapmeR-MALAT-1 at 25 mg/Kg twice a week for five treatment. Real-time tumor growth was monitored using the IVIS imaging system 200 series (PerkinElmer). Representative images of mice analysed before starting the treatment (day 0) and at different times (days 16, 24) were shown. Data were acquired and analysed using the Living Image Software version 4.3. **B** Quantitative analyses of bioluminescence signals in the liver were shown. Luminescent signals are expressed as mean of total flux of photons/sec/cm2/steradian (p/s/cm2/sr). Error bars represent SD. *p*-values were calculated using an unpaired two-tailed t-test; *p < 0.05, ** *p*-value < 0.01

**Correct Fig. 8**

**Fig. 8:**
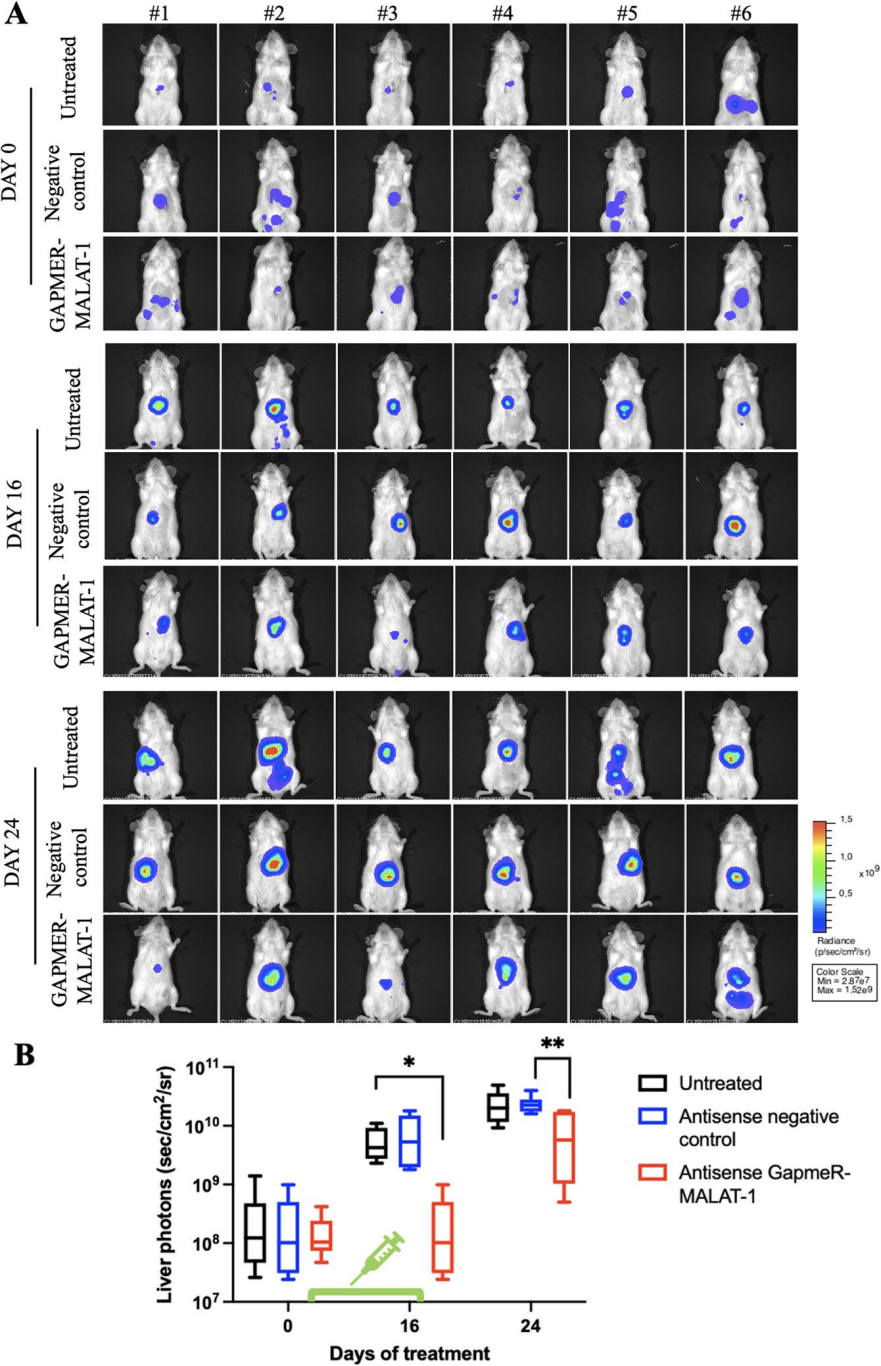
**A** Hep3B-LUC cells were injected in the liver of NOD SCID mice. Mice were treated intraperitoneally (ip) with antisense LNA negative control at 25 mg/Kg twice a week for five treatments and with antisense LNA GapmeR-MALAT-1 at 25 mg/Kg twice a week for five treatment. Real-time tumor growth was monitored using the IVIS imaging system 200 series (PerkinElmer). Representative images of mice analysed before starting the treatment (day 0) and at different times (days 16, 24) were shown. Data were acquired and analysed using the Living Image Software version 4.3. **B** Quantitative analyses of bioluminescence signals in the liver were shown. Luminescent signals are expressed as mean of total flux of photons/sec/cm2/steradian (p/s/cm2/sr). Error bars represent SD. *p*-values were calculated using an unpaired two-tailed t-test; *p < 0.05, ** *p*-value < 0.01

The correction does not compromise the validity of the conclusions and the overall content of the article. The author group has been updated above and the original article [1] has been corrected.

## References

[1] Bocchetti M, Cossu AM, Porru M, et al. MiR-423-5p is a metabolic and growth tuner in hepatocellular carcinoma via MALAT-1 and mitochondrial interaction. J Exp Clin Cancer Res. 2025;44:270. 10.1186/s13046-025-03524-2. 41029313 10.1186/s13046-025-03524-2 PMC12487375

