# Supplementary figures and images for: MiR-423-5p is a metabolic and growth tuner in hepatocellular carcinoma via MALAT-1 and mitochondrial interaction
Source: J Exp Clin Cancer Res. 2025 Sep 30;44:270. doi: 10.1186/s13046-025-03524-2 (PMC12487375; doi:10.1186/s13046-025-03524-2)

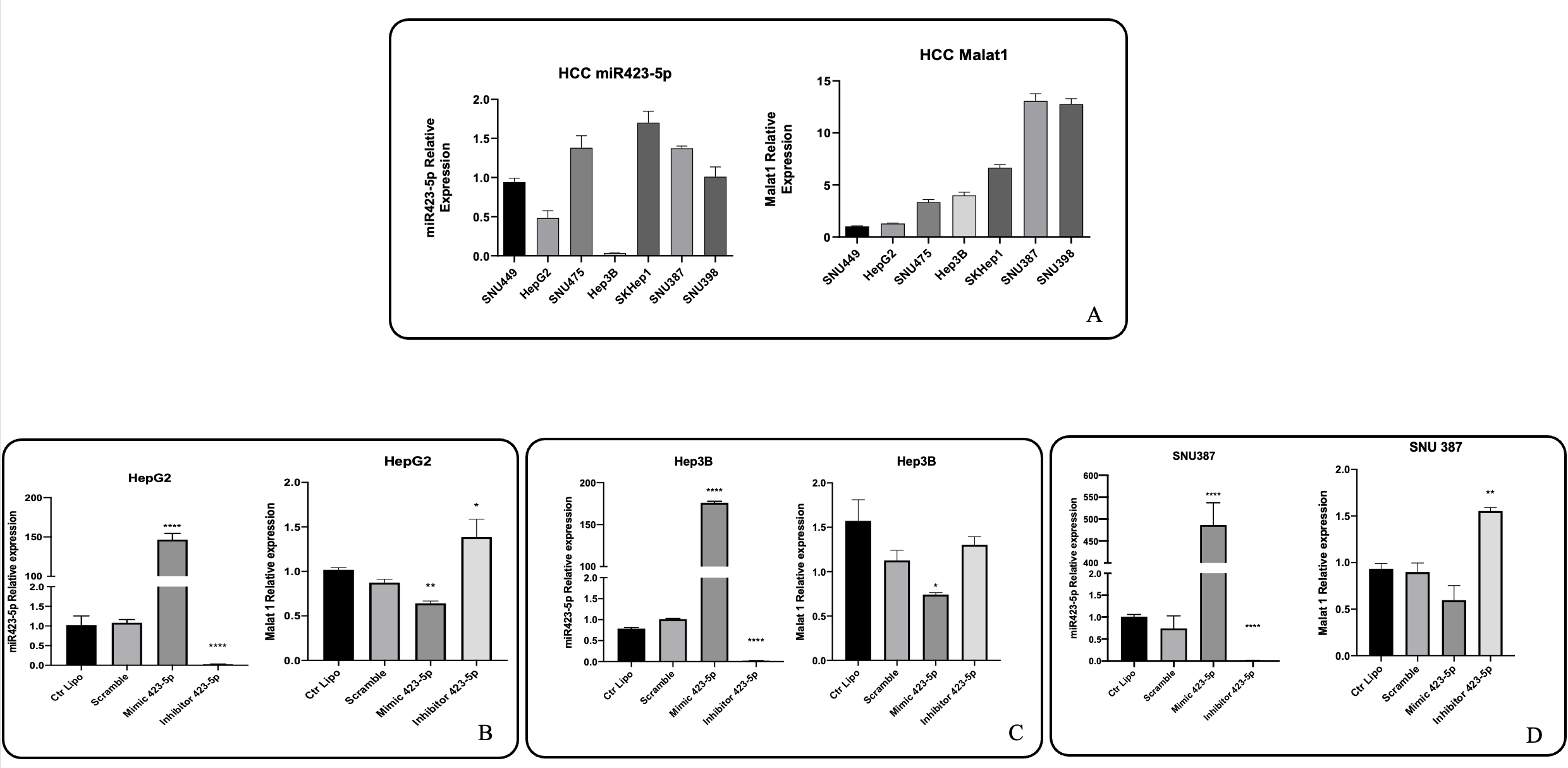

Supplement: Supplementary file 1 — Supplementary Material 1. [file 13046_2025_3524_MOESM1_ESM.zip › S1.png]

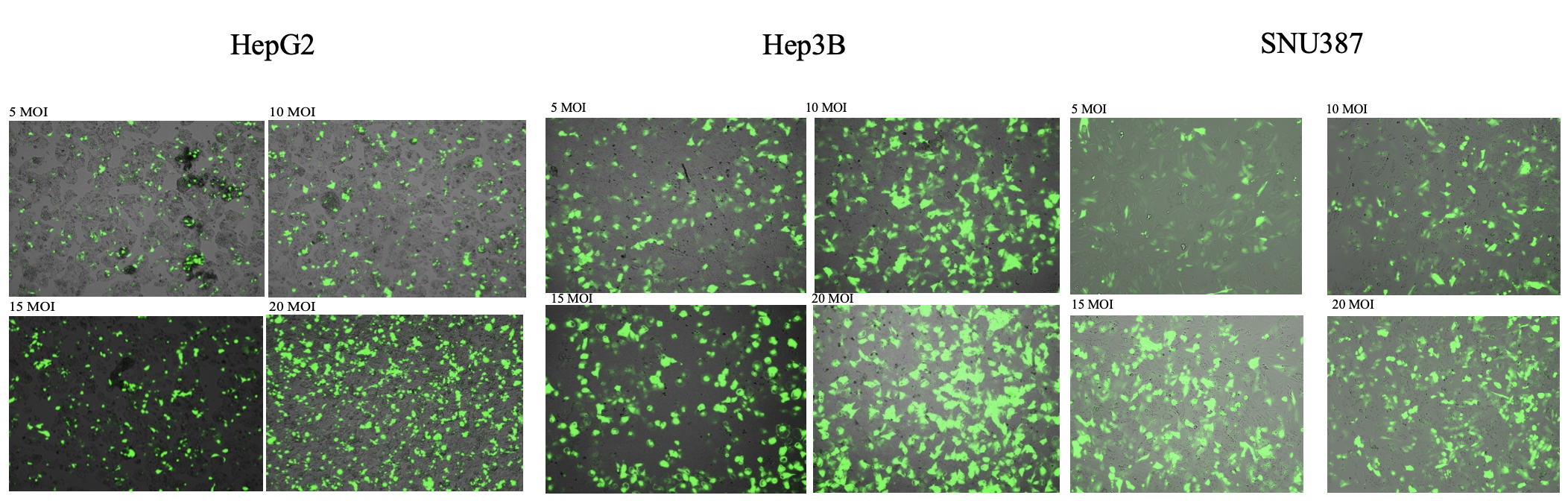

Supplement: Supplementary file 1 — Supplementary Material 1. [file 13046_2025_3524_MOESM1_ESM.zip › S2.png]

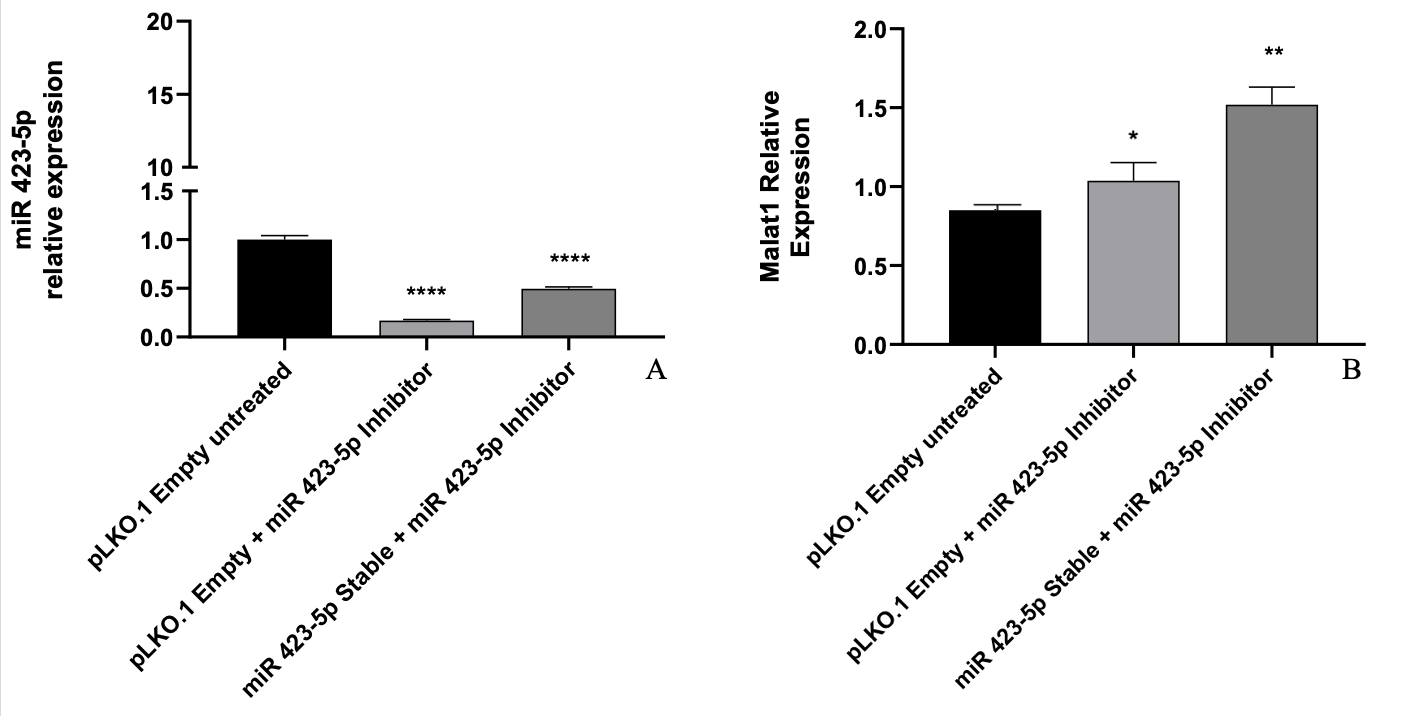

Supplement: Supplementary file 1 — Supplementary Material 1. [file 13046_2025_3524_MOESM1_ESM.zip › S3.png]

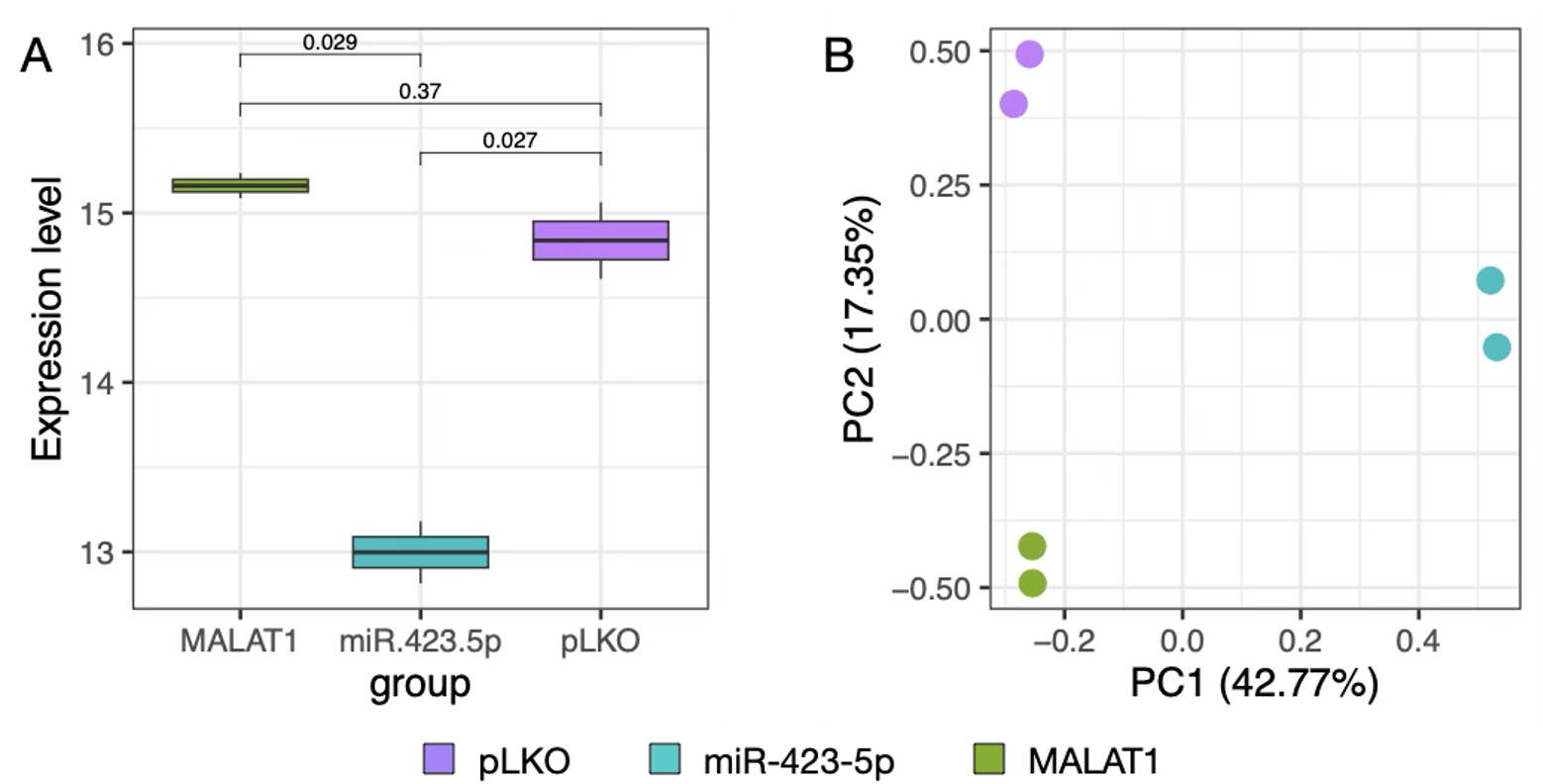

Supplement: Supplementary file 1 — Supplementary Material 1. [file 13046_2025_3524_MOESM1_ESM.zip › S4.png]
